# Atom-hybridization for synthesis of polymetallic clusters

**DOI:** 10.1038/s41467-018-06422-8

**Published:** 2018-09-24

**Authors:** Takamasa Tsukamoto, Tetsuya Kambe, Aiko Nakao, Takane Imaoka, Kimihisa Yamamoto

**Affiliations:** 1 JST-ERATO, Yamamoto Atom Hybrid Project, Institute of Innovative Research, Tokyo Institute of Technology, Yokohama, 226-8503 Japan; 2 Laboratory for Chemistry and Life Science, Tokyo Institute of Technology, Yokohama, 226-8503 Japan; 3 RIKEN, 2-1 Hirosawa, Wako, Saitama 351-0198 Japan; 4 JST-PRESTO, 4-1-8 Honcho, Kawaguchi, Saitama 332-0012 Japan

## Abstract

The chemistry of metal clusters on the sub-nanometer scale is not yet well understood because metal clusters, especially multimetallic clusters, are difficult to synthesize with control over size and composition. The template synthesis of multimetallic sub-nanoclusters is achieved using a phenylazomethine dendrimer as a macromolecular template. Its intramolecular potential gradient allows the precise uptake of metal precursor complexes containing up to eight elements on the template. The usefulness of this method is demonstrated by synthesizing multimetallic sub-nanoclusters composed of five elements (Ga_1_In_1_Au_3_Bi_2_Sn_6_). The size and composition of this cluster can be precisely controlled and the metals involved are alloyed with each other. This approach provides the ability to easily blend different metals in various combinations to create new materials on the sub-nanometer scale, which will lead to the development of a new area in the field of chemistry.

## Abstract (web summary)

Multimetallic clusters are difficult to synthesize with control over elemental composition and organization. Here, the authors use dendrimers to precisely template the formation of five-element sub-nanoclusters, providing an elegant route to otherwise-inaccessible multinary compounds.

## Introduction

Materials composed of three or more metal elements, known as multinary compounds, are useful for applications such as superconducting materials^1,2^, semiconductor solar cell devices^3,4^, metallic glasses^5,6^, and catalysts^7–9^. They possess unique properties that cannot be obtained from a single element and thus are highly attractive targets. The short supply of rare metal elements limits their applications, and increases interest in the development of materials that maximize their utility. However, the available combinations of elements for multinary compounds are limited. Metal elements that have thermodynamically unfavorable alloy formation at room temperature often are phase-separated at bulk state and exhibit unreliable performance of the individual elements. Although miniaturization of structures of these compounds into a nanometer scale often allows us to blend such metal elements uniformly, it has been reported that a combination of more than three different metals tends to involve metal segregation in some segments even for nanosized clusters^9–17^. Therefore, it is necessary for development of functional multinary compounds to overcome this limitation of combination of blendable metals.

**Fig. 1:**
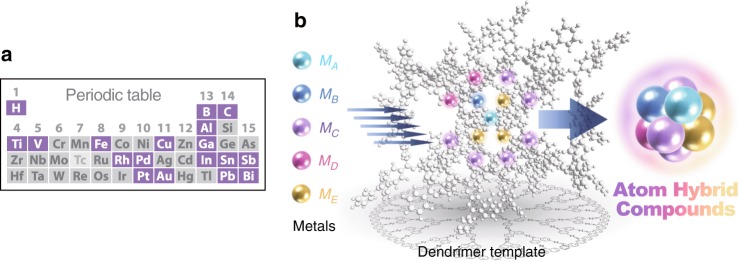
Template synthesis of multimetallic sub-nanoclusters. **a** Metal elements precisely accumulated on the the phenylazomethine (DPA) dendrimer (purple-colored) and **b** concept of the “atom hybridization” method

In contrast, a sub-nanosized cluster (2–30 atoms) with no phase-segregated state on the atomic level would be alloyed, making chemical bonds between the different metals, even if they were separated in the bulk or nanocluster state (Supplementary Fig. 1). An increase in mixing entropy due to disordered phases in its atomic level structure and a decrease in mixing enthalpy due to limited number of nearest neighbor atoms are expected to prevent phase-separation. Innovative materials are possible if metal elements can be blended on a sub-nanometer scale, and the multinary sub-nanoclusters could possess entirely new properties by hybridization of their atomic orbitals, which is most effective in a quantum-sized region. The present report describes this new concept, called “atom hybridization.” However, sub-nanoclusters composed of many kinds of metals have been impossible to synthesize because their size and composition cannot be accurately controlled using conventional techniques. To overcome this obstacle, macromolecular ligand templates were investigated to promote the synthesis of multimetallic sub-nanosized materials. A dendrimer with phenylazomethine moieties (DPA) was used as a template modified for the synthesis of monodispersed sub-nanoclusters^18–22^. The structure of DPA contains imine units as coordination sites for the metal precursors, and the dendritic DPA induces an intramolecular potential gradient promoting layer-by-layer stepwise complexation of the metal salts from the inner imines to the outer ones. This characteristic function cannot be realized by general dendrimers, such as polyamidoamine (PAMAM), which do not produce the potential gradient^23–28^. By taking advantage of dendritic DPA, the precise accumulation of various metal salts on the template was accomplished (Fig. 1a and Supplementary Figs. 2–20). This distinctive complexation behavior allows adjustment of the accumulation number and ratio of the different metal salts on the template^21^, leading to precise control of cluster size and composition on the sub-nanometer scale. The present report describes the synthesis of multimetallic sub-nanoclusters, employing a DPA dendrimer whose potential gradient is modulated by a change in the core structure (Fig. 1b).

## Results and discussion

### Precise multimetallic accumulation on the dendrimer

**Fig. 2:**
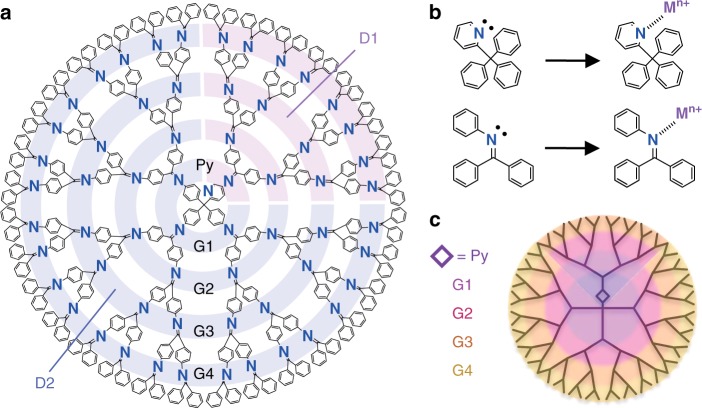
The DPA dendrimer template. **a** Molecular structure of the generation 4 dendrimer (PyTPM-G4). **b** Complexation of metal salts at the imine sites of pyridine and DPA. **c** Potential gradient on PyTPM-G4. (purple: higher electron density, yellow: lower electron density). Py pyridine, G generation, and D dendron

When different metal salts were added to the DPA dendrimers, experiments confirmed that metal salts possessing stronger Lewis acidity were coordinated at imine sites possessing stronger Lewis basicity on inner generation layers, and weaker acidic metal salts were coordinated at weaker basic imines on outer generation layers^21^. Application of this principle allowed the precise synthesis of multimetallic sub-nanoclusters by accumulating metal salts while controlling the Lewis acidity of the metal salts and Lewis basicity of the imines. A generation 4 dendrimer with a phenylazomethine skeleton and a mono(2-pyridyl)-triphenylmethane core (PyTPM-G4) was used as a template for accumulation of the metal salts^18,19,21,29^ (Fig. 2a). The pyridyl-substituted core provided a dendrimer template with a characteristic potential gradient. The PyTPM-G4 dendrimer has 61 imine sites at the pyridine (1) and phenylazomethine (60) moieties that act as coordination sites for metal salts (Fig. 2b). Four types of electrophilic nitrogen atoms exist on its dendron that provide the potential gradient across generations 1–4 (Fig. 2c). The imine sites on the inner generation layers are more strongly basic than those on the outer ones, allowing attachment of metal salts in order of the generation number. In addition, the basicity of the imines on the phenylazomethine moieties also differs depending on the aromatic rings at the core (benzene and pyridine). Using this behavior, metal salts can be arranged precisely and complexly due to both the generation number and the molecular structure of the root of the dendron on PyTPM-G4. The pyridine moiety at the core plays a dual role as both a coordination site and controller of the basicity of the imines on the phenylazomethine dendrons. The Lewis basicity at the imine sites on PyTPM-G4 were separated into nine sections, with the order of basicity as N_Py_ (1) > N_D1-G1_ (1) > N_D2-G1_ (3) > N_D1-G2_ (2) > N_D2-G2_ (6) > N_D1-G3_ (4) > N_D2-G3_ (12) > N_D1-G4_ (8) > N_D2-G4_ (24) from the complexation behavior with Ga^III^Cl_3_ as a Lewis acid^29^ (Fig. 2a).

To extend the list of available metals, the stepwise complexation of other metal salts (In^III^Br_3_, Au^III^Cl_3_, Bi^III^Cl_3_, and Sn^II^Br_2_) on PyTPM-G4 was investigated. The characteristic complexation behavior of metal salts with the imines could be monitored by UV-Vis absorption spectrophotometry. The results indicated that other metal salts produced absorption spectral changes similar to those produced by Ga^III^Cl_3_ (Supplementary Fig. 21). For example, for In^III^Br_3_, the absorption of free imine sites on the dendrimer decreased while the absorption of the metal-imine complexes remained on the spectra upon addition of In^III^Br_3_ to a solution of the dendrimer in 1:1 dichloromethane/acetonitrile (DCM/AN) (Supplementary Fig. 22A). During addition of In^III^Br_3_, five changes in the position of the isosbestic point were observed at 342.0, 350.0, 363.5, 365.0, 366.0 nm with amounts of 1, 2, 5, 7, and 13 equiv. In^III^Br_3_ on PyTPM-G4, respectively (Supplementary Fig. 22B). This result indicates that the location of accumulation of In^III^Br_3_ was based on the basicity of the imine sites determined by the potential gradient on PyTPM-G4 (Supplementary Fig. 22C). Under the same conditions, stepwise accumulation of 1, 2, 5, 7, and 13 equiv. of other metals (Au^III^Cl_3_, Bi^III^Cl_3_, and Sn^II^Br_2_) could also be controlled along with Ga^III^Cl_3_ and In^III^Br_3_, as determined by the spectral changes in the UV-Vis absorption spectra and the changes in the position of the isosbestic point (Supplementary Figs. 23–25).

The coordination strength of these metal salts to the imine site was investigated using the generation 1 dendrimer TPM-G1 as a control compound with four equivalent imine sites (Supplementary Fig. 2B). The strength of the coordination of metal salts determines their order of accumulation and so is important for precise multimetallic accumulation on a dendrimer. For all metal salts, the UV-Vis absorption spectra of TPM-G1 changed upon titration due to complexation of the imines along with PyTPM-G4 (Supplementary Fig. 26A). According to the titration curves of TPM-G1, the order of coordination strength for the metal salts was: Ga^III^Cl_3_ > In^III^Br_3_ > Au^III^Cl_3_ > Bi^III^Cl_3_ > Sn^II^Br_2_ (Supplementary Fig. 26B). Differences in the binding constant of two metal salts were estimated to be on the order of 0.5–1 order of magnitude, determined from theoretical titration curves (Supplementary Fig. 27). This difference is large enough to suppress the exchange of metal salts between imine sites with different Lewis basicity.

**Fig. 3:**
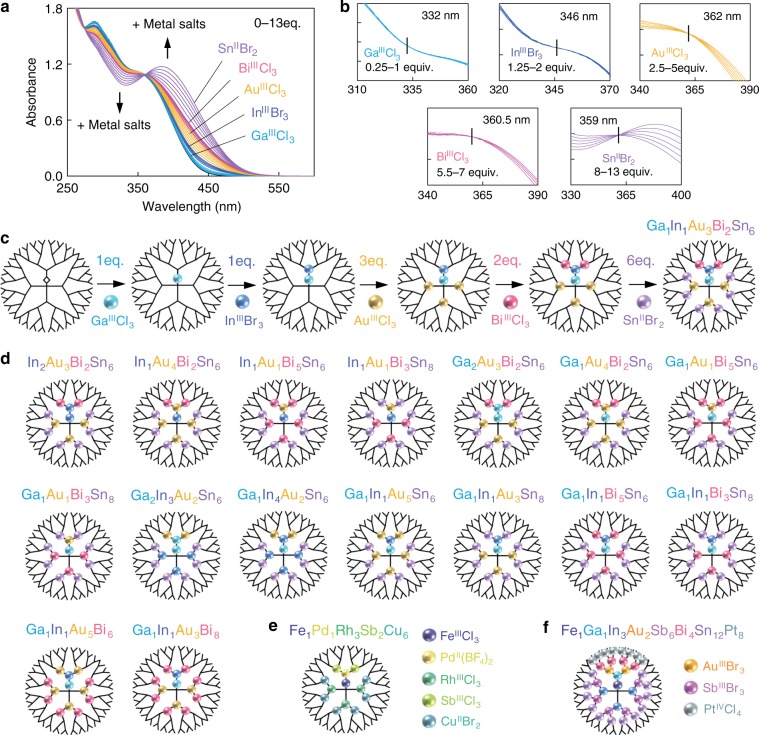
Multimetallic accumulation on a dendrimer template. **a** Changes in UV-Vis absorption spectra of PyTPM-G4 upon addition of 1 equiv. Ga^III^Cl_3_, 1 equiv. In^III^Br_3_, 3 equiv. Au^III^Cl_3_, 2 equiv. Bi^III^Cl_3_, and 6 equiv. Sn^II^Br_2_ in 1:1 DCM/AN. **b** Isosbestic points during complexation with metal salts in layers 0–1, 1–2, 2–5, 5–7, and 7–13. **c** Layer-by-layer stepwise multimetallic accumulation of metal salts on PyTPM-G4. **d** Diversity of multimetallic accumulation of four elements at amounts up to 13 equiv. **e** Multimetallic accumulation of five elements (Fe^III^Cl_3_, Pd^II^(BF_4_)_2_, Rh^III^Cl_3_, Sb^III^Cl_3_, and Cu^II^Br_2_) at amounts up to 13 equiv. on PyTPM-G4. **f** Multimetallic accumulation of eight elements (Fe^III^Cl_3_, Ga^III^Cl_3_, In^III^Br_3_, Au^III^Br_3_, Sb^III^Br_3_, Bi^III^Cl_3_, Sn^II^Br_2_, and Pt^IV^Cl_4_) at amounts up to 37 equiv. on PyTPM-G4

Next, five types of metal salts were accumulated on one molecular PyTPM-G4 template. This multimetallic accumulation used the difference in Lewis acidity among the metal salts and the Lewis basicity among the imine sites on PyTPM-G4. The metal salts, Ga^III^Cl_3_, In^III^Br_3_, Au^III^Cl_3_, Bi^III^Cl_3_, and Sn^II^Br_2_ were added to PyTPM-G4 in descending order of coordination strength. The UV-Vis absorption spectra were recorded during titration of the metal salts in the order: 1 equiv. Ga^III^Cl_3_, 1 equiv. In^III^Br_3_, 3 equiv. Au^III^Cl_3_, 2 equiv. Bi^III^Cl_3_, and 6 equiv. Sn^II^Br_2_ to PyTPM-G4 (Fig. 3a). Five changes were observed in the position of the isosbestic point upon each addition of metal salts during the titration (Fig. 3b). This result indicates that the locations of accumulation of the metal salts was not changed upon subsequent addition of other metal salts, confirming that stronger and weaker Lewis acidic metal salts were preferentially coordinated at stronger and weaker Lewis basic imine sites of PyTPM-G4, respectively (Fig. 3c). This multimetallic accumulation technique could also be utilized for other metal species such as Fe, Pd, Rh, Sb, Cu, and Pt (Fig. 3e and Supplementary Figs. 28 and 29). In addition, the accumulation of four metal elements in various combinations without any changes in the number of atoms (Fig. 3d) demonstrated the utility of PyTPM-G4 template. Changes in the UV-Vis absorption spectra during titration on PyTPM-G4 are shown in Supplementary Figs. 30A-45A. For all combinations, five changes occurred in the position of the isosbestic point in the spectra, along with those of the five-element (Supplementary Figs. 30B-45B), indicating that the vast number of metal and imine position combinations will allow fine-tuning of the chemical composition in a sub-nanocluster with a certain atomicity. Furthermore, the accumulation of other metals on a dendrimer was also investigated and showed that modulating the coordination strengths of the metal salts by changes in their counter anions, the multimetallic accumulation composed of six to eight metal elements was accomplished (Fig. 3f and Supplementary Figs. 46–48).

### Template synthesis of multimetallic sub-nanoclusters

**Fig. 4:**
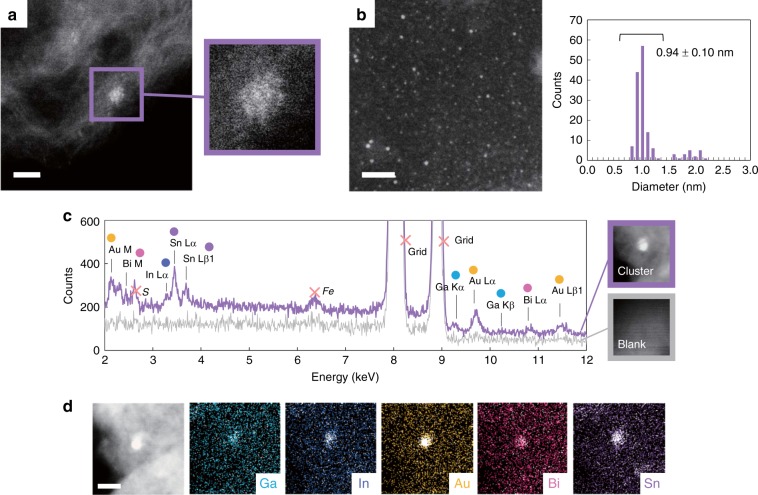
STEM and EDS measurements of five-element sub-nanoclusters. **a** STEM image of five-element sub-nanocluster supported on KB (scale bar: 2 nm) (left) and an enlarged view of the cluster (right). **b** STEM image of the clusters supported on a carbon substrate (scale bar: 20 nm) (left) and a histogram of cluster size distributions (right). **c** STEM/EDS analysis of five-element sub-nanocluster on KB and blank. Peaks of S and Fe are originated from slight impurities of KB. **d** STEM/EDS mapping of the cluster on KB (scale bar: 5 nm)

Precise synthesis of the five-element sub-nanoclusters was investigated using PyTPM-G4 as a template. The five metal salts-dendrimer complex (Ga^III^Cl_3_)_1_(In^III^Br_3_)_1_(Au^III^Cl_3_)_3_(Bi^III^Cl_3_)_2_(Sn^II^Br_2_)_6_@PyTPM-G4 was prepared in 1:1 DCM/AN. The metal salts on the dendrimer were reduced by NaBH_4_, based on reduction potentials, and produced five-element sub-nanoclusters supported on carbon (Ketjen Black (KB)). The multimetallic cluster was confirmed by high-resolution scanning transmission electron microscopic (STEM) images (Fig. 4). According to atomic resolution observation at high magnification of the STEM image, the sub-nanoclusters were composed of more than a dozen atoms that could be observed directly by atom counting (Fig. 4a). In contrast, STEM observations at low magnification revealed that the multimetallic clusters were obtained in high yield without any large nanoclusters (Fig. 4b). The histogram of the cluster size distribution showed that these sub-nanoclusters are almost monodispersed (standard deviation: ± 0.10 nm). Slightly larger clusters were also barely observed besides these sub-nanoclusters due to their aggregation. Their size was ~0.9–1.0 nm, which is consistent with the theoretical size of the clusters estimated by DFT calculations^30^ (Supplementary Fig. 49).

The constitutive components of the sub-nanoclusters were confirmed by energy dispersive X-ray spectrometry with STEM (STEM/EDS). The STEM/EDS analysis was conducted on slightly larger sub-nanoclusters (Fig. 4b) because analysis of smaller ones was difficult due to low sensitivity and occasional sub-nanocluster collapse caused by the electron beam. Thus, spectral peaks attributed to Ga, In, Au, Bi, and Sn were detected in the field of view where the cluster existed on KB (Fig. 4c). In contrast, no peaks for these elements were observed in the region without the cluster in the same sample, indicating that the sub-nanoclusters were composed of at least five metals accumulated on PyTPM-G4. This result was also supported by STEM/EDS mapping, in which these five elements were detected only on the image of the cluster (Fig. 4d).

The synthesis of sub-nanosized five-element clusters is possible only through the use of the dendrimer templates described here. Without using the dendrimer template, aggregation of the clusters occurred along with an increase in cluster size (>10 nm) (Supplementary Fig. 50A). The STEM/EDS analysis showed that gold and bismuth become separated and covered by tin in the aggregated large clusters (Supplementary Fig. 50B and C). These results indicate that synthesis using the dendrimer template allowed control of cluster size, and suppressed aggregation of the clusters and alloying of the metals.

**Fig. 5:**
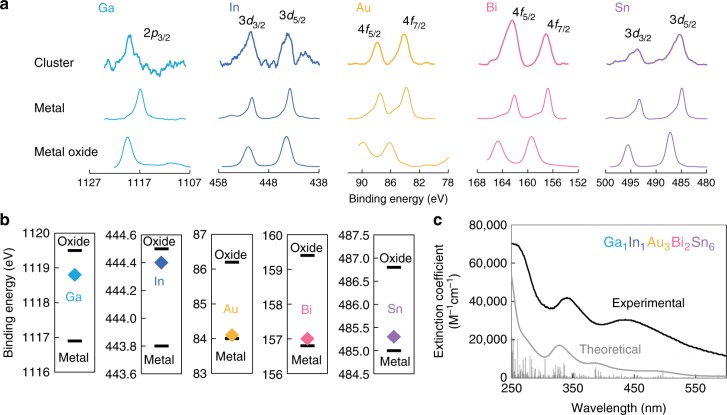
XPS and UV-Vis absorption data for five-element sub-nanoclusters. **a** XPS spectra of five-element clusters on a glassy carbon substrate, metals, and metal oxides focusing on the Ga 2*p*_3/2_, In 3*d*, Au 4*f*, Bi 4*f*, and Sn 3*d* core levels. **b** Binding energy of the five-element cluster, metals, and metal oxides for each element (Ga 2*p*_3/2_, In 3*d*_5/2_, Au 4*f*_7/2_, Bi 4*f*_7/2_, and Sn 3*d*_5/2_). **c** UV-Vis absorption spectrum of the five-element cluster with PyTPM-G4 in 1:1 DCM/AN, and a theoretical spectrum estimated from the TD-DFT calculations. Absorption of PyTPM-G4 was canceled by the reference solution

X-ray photoelectron spectroscopy (XPS) focusing on the Ga 2*p*_3/2_, In 3*d*, Au 4 *f*, Bi 4*f*, and Sn 3*d* core levels detected a signal for each element in the five-element sub-nanocluster on a glassy carbon substrate, bulk metal, and metal oxide (Fig. 5a). The calculated ratio of Ga:In:Au:Bi:Sn of the cluster were 1:0.92:3.4:1.7:6.1, which agreed well with the theoretical values (Ga:In:Au:Bi:Sn = 1:1:3:2:6). Spectral peaks for some elements in the cluster were shifted to a slightly higher binding energy compared to the bulk metal (0) (Fig. 5b). The degree of the shift was associated with the element electronegativity (EN) (EN: Ga, In = ca. 1.8, Bi, Sn = ca. 2.0, Au = ca. 2.5)^31^. The dependence of the charge distribution on the EN suggests that these elements are hybridized in the cluster. The ratio of the constitutive elements did not depend on the microscopic X-ray irradiated positions on the substrate (Supplementary Fig. 51).

According to DFT calculations, the five-element sub-nanocluster had absorption bands in the UV-Vis range. An optimized molecular structure of the cluster is shown in Supplementary Fig. 49. The cluster solution was a light orange color and its absorption spectrum could be experimentally observed in situ, although the light scattering by solid by-products derived from NaBH_4_ were superimposed on the spectra (Fig. 5c and Supplementary Fig. 52B). The spectra need to be recorded within a few minutes after sub-nanocluster synthesis to avoid aggregation. As a result, the spectral shapes, absorption wavelength, and extinction coefficient agreed well between the theoretical and experimental absorption spectra. In contrast, the characteristic spectrum was not observed for monometallic clusters (Ga_1_, In_1_, Au_3_, Bi_2_, and Sn_6_) or for clusters synthesized without using the dendrimer template (Supplementary Fig. 52A). The spectral peak at 440 nm on the experimental one is expected to be originated from interactions of the sub-nanocluster with the imines on the dendrimer or the nitriles in the solvents.

For constitutive components containing more than five metal elements, the EDS or XPS analysis of the clusters tended to be difficult due to overlap of the signals for each element. However, synthesis of another five-element sub-nanocluster Fe_1_Pd_1_Rh_3_Sb_2_Cu_6_ composed of metals different from the cluster described above was successful (Supplementary Figs. 53, 54). Moreover, EDS analysis of the six-element cluster Ga_1_In_1_Au_3_Bi_2_Sn_6_Pt_4_ was conducted to demonstrate the synthesis of multimetallic sub-nanoclusters with metals from additional classes. After preparation of the multimetallic cluster along with the five-element cluster (Supplementary Fig. 46), the appearance of sub-nanoclusters of the corresponding size was confirmed by STEM (Supplementary Fig. 55A), while STEM/EDS analysis revealed that the main elements (Ga, In, Au, Bi, Sn, and Pt) were detected only in the image of the sub-nanocluster (Supplementary Fig. 55B, C). These results indicate that multimetallic sub-nanoclusters composed of more than five metal elements can be obtained by the dendrimer template synthesis method.

Overall, the template synthesis of multimetallic sub-nanosized clusters, which cannot be obtained by statistical approaches, was demonstrated without the use of complicated synthetic methods. The method will be also available for suitably designed other dendritic systems such as dendrimer templates with some coordinating units possessing different basicity. This approach using the dendrimers as a nanosynthesizer should become a key method for investigating the unexplored area of chemistry on a sub-nanometer scale. As one of the application potentialities of this result, advanced catalytic materials can be realized by applying both two enhancement factors of catalytic ability: the miniaturization of its structure^19^ and the alloying of different metals^22^.

## Methods

### Materials

PyTPM-G4 and TPM-G1 were synthesized according to a previous reports^28^. Dichloromethane (DCM), acetonitrile (AN), and methanol were purchased from Kanto Chemical Co., Inc. All solvents were anhydrous grade. Au^III^_2_O_3_, Ga^III^Cl_3_, Rh^III^Cl_3_(aq)_3_, and Sb^III^Br_3_ were purchased from Alfa Aesar. Ga^0^, Ga^III^_2_O_3_, In^0^, In^III^_2_O_3_, Au^0^, Bi^0^, Bi^III^_2_O_3_, Sn^0^, Sn^II^O, Sn^IV^O_2_, Pt^0^, Au^III^Cl_3_, Au^III^Br_3_, Bi^III^Cl_3_, Cu^II^Br_2_, In^III^Br_3_, Fe^III^Cl_3_, Pd^II^(an)_4_(BF_4_)_2_, Pt^IV^Cl_4_, Sb^III^Cl_3_, and Sn^II^Br_2_ were supplied by Sigma-Aldrich Co. LLC. NaBH_4_ was purchased from Wako Pure Chemical Industries, Ltd. Ketjen Black (KB) was obtained from Lion Specialty Chemicals Co., Ltd.

### Instruments

The UV-vis absorption spectra were obtained by Shimadzu UV-3100, UV-3150 and UV-3600 spectrometers. STEM measurements were carried out by a JEOL JEM-ARM200F equipped with an EDS analyzer (acceleration voltage: 80 kV). XPS was recorded by a Shimadzu ESCA-3400 with Mg Kα (12 kV, 30 mA) using as an X-ray source. The binding energy was standardized using the Pt 4 *f* peak at 71.0 eV for Au-containing clusters and Au 4 *f* peak at 84.0 eV for other samples. XPS measurements for position dependence were carried out with VG ESCALAB 250 spectrometer (Thermo Fisher Scientific K.K.), using monochromatized Al Kα X-ray radiation (1486.6 eV). The system was operated at 15 kV and 200 W. The base pressure of the analysis chamber was less than 10^−8^ Pa. The pass energy was 50.0 eV (wide scan) and 20 eV (individual narrow scan). These characterization were carried our using transfer vessel to avoid moisture/air exposure of the samples. Standardization was achieved using the C 1 *s* transition (284.8 eV). Background removal was carried out with the Thermo Electron Advantage analysis software package.

### Titration method for PyTPM-G4 on UV-vis absorption spectra

A 3.0 × 10^–6^ M PyTPM-G4 solution (DCM/AN = 1:1, 3 mL, 20 °C) was prepared in a 1 cm optical quartz cell. 3.0 × 10^–3^ M metal salts in an AN solution was added to a 3 mL solution of PyTPM-G4 in a stepwise fashion from 0 to 13 equiv. of a dendrimer molecule (0, 0.25, 0.5, 0.75, 1, 1.25, 1.50, 1.75, 2, 2.5, 3.0, 3.5, 4.0, 4.5, 5.0, 5.5, 6.0, 6.5, 7, 8, 9, 10, 11, 12, and 13 equiv.).

### Titration method for TPM-G1 on UV-vis absorption spectra

A 3.0 × 10^–5^ M TPM-G1 solution (DCM/AN = 1:1, 3 mL) was prepared in a 1 cm optical quartz cell. 9.0 × 10^–3^ M metal salts in AN solution was added to a 3 mL solution of PyTPM-G4 in a stepwise fashion up to saturation of the spectral change.

### Reduction of metal salts accumulated on PyTPM-G4

The preparation method of the complexes of PyTPM-G4 and the metal salts was the same as those for the UV-vis absorption measurement mentioned above. Sixty equiv. of NaBH_4_ (300 mM, MeOH) on the accumulated metal salts was added to the complex solution with stirring under an inert atmosphere (Ar).

### Sample preparation of clusters for STEM measurement

The solution of the clusters with PyTPM-G4 was prepared by reduction of accumulated metal salts as mentioned above. For the high-resolution STEM observation, a suspension of KB (DCM/AN = 1:1, 1 mL) was added to the reaction mixture and stirred for 1.5 h. The sub-nanocluster with dendrimer on KB in solution was dropped onto a copper grid with a carbon film and dried under overnight vacuum. For the STEM observation at low magnification, the sub-nanocluster with the dendrimer without KB in solution was dropped onto a copper grid with a carbon substrate and dried overnight under vacuum.

### Sample preparation of clusters for XPS measurement

The solution of clusters with PyTPM-G4 was prepared by the same method described in STEM measurement section. A 5 μL portion of the sub-nanocluster solution with a dendrimer was then dropped 10 times (total 50 μL) onto a glassy carbon substrate (5 mm × 5 mm). After drying, the XPS analysis was conducted as soon as possible. All operations were carried out under an inert atmosphere. The samples were subjected to Ar sputtering (2 kV, 30 mA) for 6 min to remove any surface impurities before the measurement.

### Sample preparation of clusters for UV-vis absorption measurement

The solution of clusters with PyTPM-G4 was obtained in a 1 cm optical quartz cell by the same method described in STEM measurement section. The absorption of PyTPM-G4 was canceled by the reference solution prepared by the same preparation method as the sample except for the addition of metal precursors.

## Electronic supplementary material

Supplementary Information

## Data Availability

The data that support the findings of this study are available from the corresponding author upon reasonable request.
