# Supplementary material for: Atom-hybridization for synthesis of polymetallic clusters
Source: Nat Commun. 2018 Sep 24;9:3873. doi: 10.1038/s41467-018-06422-8 (PMC6155219; doi:10.1038/s41467-018-06422-8)
Supplement: Supplementary file 1 — Supplementary Information [file 41467_2018_6422_MOESM1_ESM.pdf]

Supplementary Information for

Atom-hybridization for synthesis of polymetallic clusters

Tsukamoto *et al.*

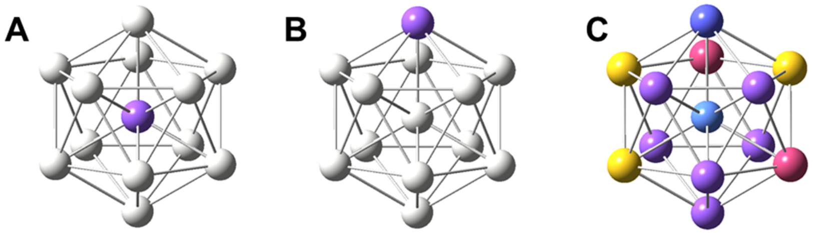

Supplementary Figure 1
The schematic representations of sub-nanosized 13-atom icosahedral cluster.
It is important for alloying or blending different metals in the sub-nanocluster that these metals make chemical bonds each other (their atomic orbitals are hybridized). Especially in sub-nanocluster composed of a dozen atoms, the different metals must theoretically make the heterometallic bonds because of its atomic level structure. For example, in the case of the sub-nanosized 13-atom one, constitutive atoms (colored atom) can make up to 13 bonds (A) or at least 6 bonds (B) with adjacent atoms. This property be also applied for the 5-elements in the 13-atom cluster and they must form chemical bonds each other (C).

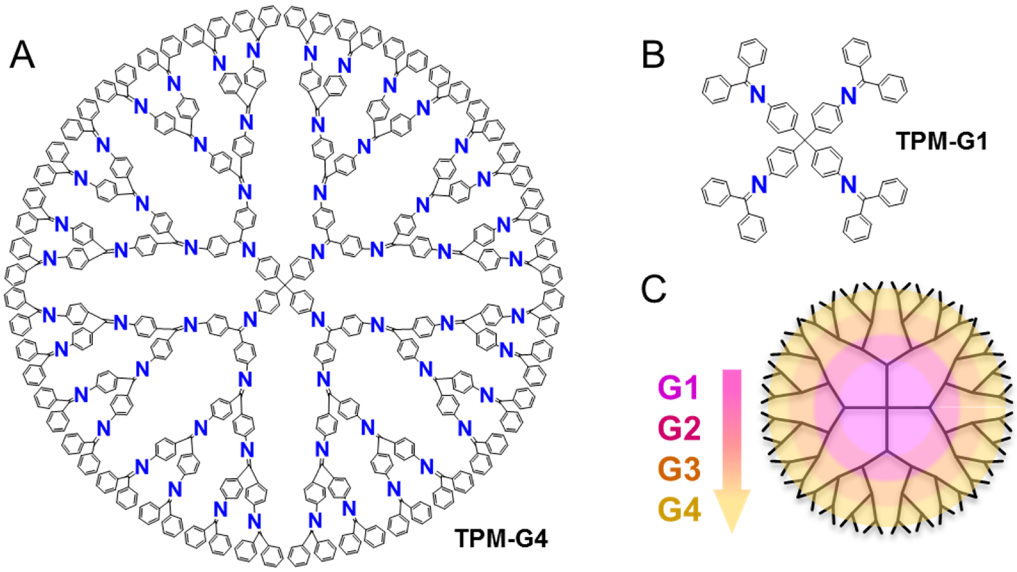

Supplementary Figure 2

The DPA dendrimer template with tetraphenylmethane core. (A) The molecular structures of the 4th generation dendrimer (TPM-G4) and (B) the 1th generation dendrimer as a model compound (TPM-G1). (C) The potential gradient on TPM-G4. (purple: higher electron density, yellow: lower electron density).

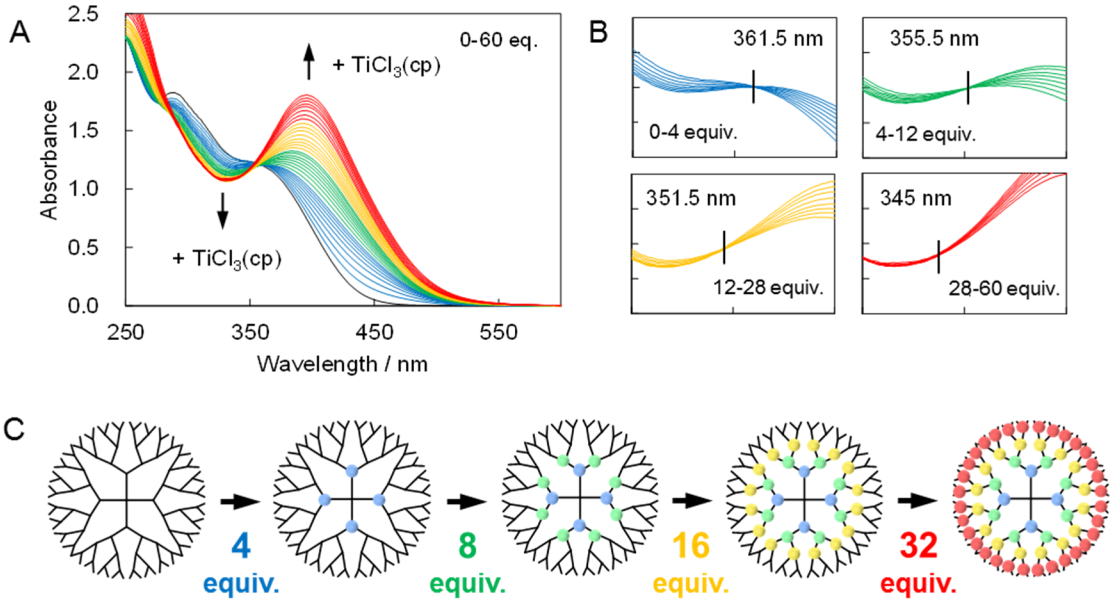

Supplementary Figure 3

Accumulation of Ti on TPM-G4. (A) Change in UV-vis absorption spectra of TPM-G4 in DCM/THF = 2:1 upon the addition of $Ti^{IV}Cl_3(cp)$ in THF up to 60 equiv. ([TPM-G4] = ca. 3.0×10^{-6} M, 20 °C, under N_2). (B) Isosbestic points during complexation with $Ti^{IV}Cl_3(cp)$ in respective layers 0–4, 4–12, 12–28 and 28–60 equiv. (C) Layer-by-layer stepwise accumulation of a metal salt on TPM-G4.

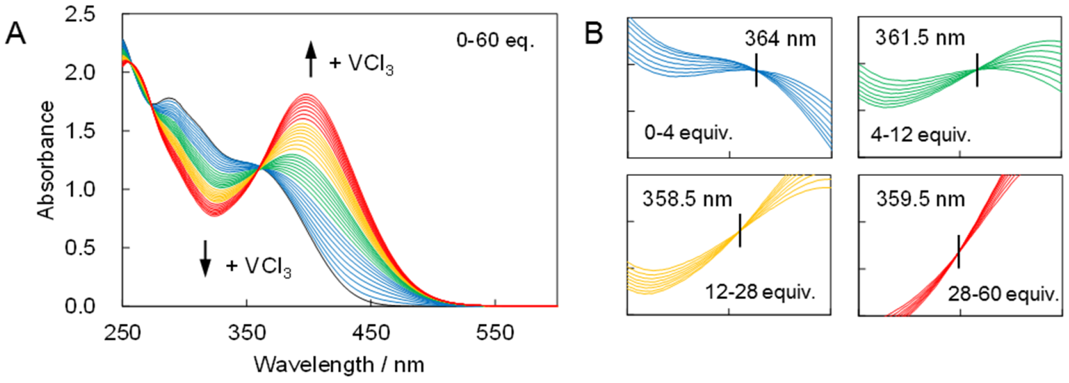

Supplementary Figure 4

Accumulation of V on TPM-G4. (A) Change in UV-vis absorption spectra of TPM-G4 in CLF/AN = 1:1 upon the addition of $V^{III}Cl_3$ in AN up to 60 equiv. ([TPM-G4] = ca. 3.0×10^{-6} M, 0 °C, under N_2). (B) Isosbestic points during complexation with $V^{III}Cl_3$ in respective layers 0–4, 4–12, 12–28 and 28–60 equiv.

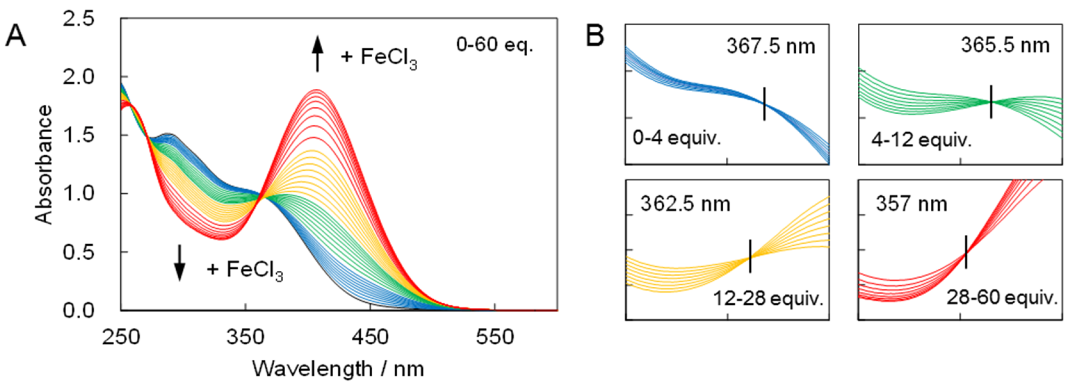

Supplementary Figure 5

Accumulation of Fe on TPM-G4. (A) Change in UV-vis absorption spectra of TPM-G4 in CLF/AN = 5:7 upon the addition of $Fe^{III}Cl_3$ in AN up to 60 equiv. ([TPM-G4] = ca. 3.0 × 10^{-6} M, 20 °C). (B) Isosbestic points during complexation with $Fe^{III}Cl_3$ in respective layers 0–4, 4–12, 12–28 and 28–60 equiv.

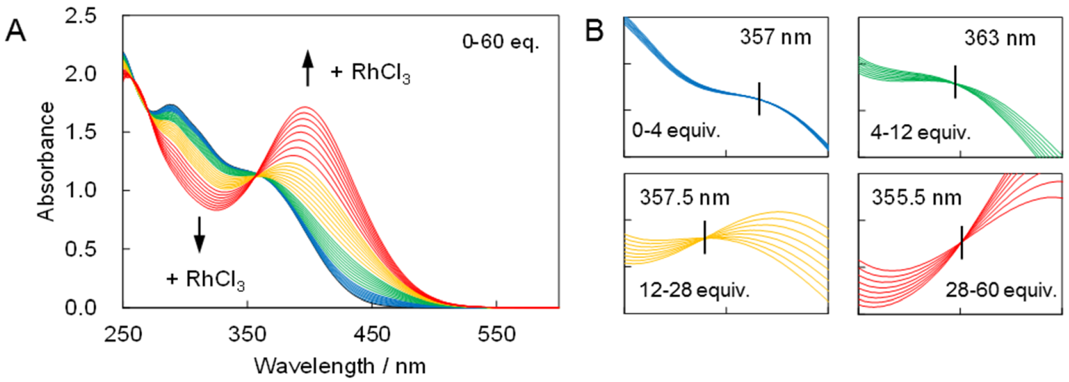

Supplementary Figure 6

Accumulation of Rh on TPM-G4. (A) Change in UV-vis absorption spectra of TPM-G4 in DCM/AN = 1:1 upon the addition of $Rh^{III}Cl_3(aq)_3$ in AN/MeOH = 250:1 up to 60 equiv. ([TPM-G4] = ca. 3.0×10^{-6} M, 20 °C). (B) Isosbestic points during complexation with $Rh^{III}Cl_3(aq)_3$ in respective layers 0–4, 4–12, 12–28 and 28–60 equiv.

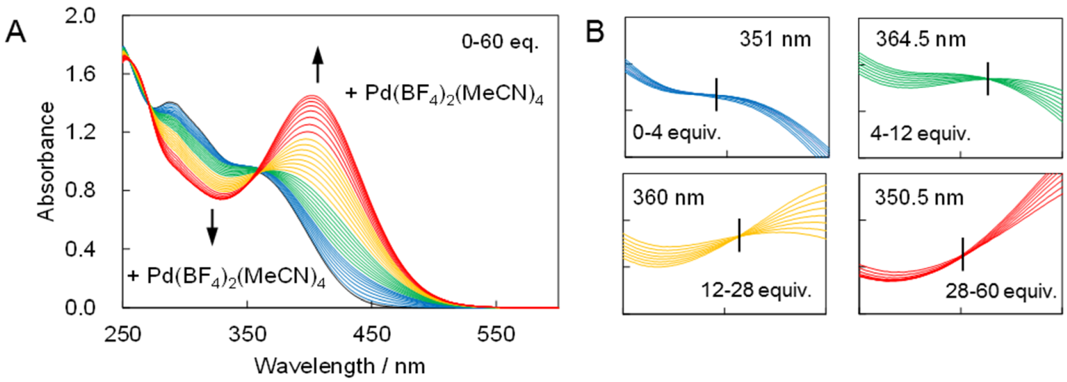

Supplementary Figure 7

Accumulation of Pd on TPM-G4. (A) Change in UV-vis absorption spectra of TPM-G4 in CLF/AN = 5:7 upon the addition of $Pd^{II}(BF_4)_3(an)_4$ in AN up to 60 equiv. ([TPM-G4] = ca. 3.0×10^{-6} M, 20 °C). (B) Isosbestic points during complexation with $Pd^{II}(BF_4)_3(an)_4$ in respective layers 0–4, 4–12, 12–28 and 28–60 equiv.

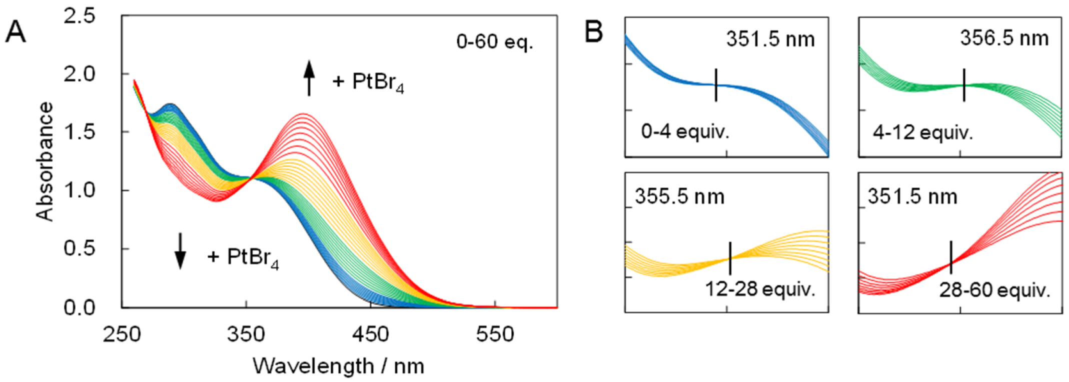

Supplementary Figure 8
Accumulation of Pt on TPM-G4. (A) Change in UV-vis absorption spectra of TPM-G4 in DOX/DMF = 40:1 upon the addition of $Pt^{IV}Br_4$ in AN up to 60 equiv. ([TPM-G4] = ca. 3.0×10^{-6} M, 20 °C). (B) Isosbestic points during complexation with $Pt^{IV}Br_4$ in respective layers 0–4, 4–12, 12–28 and 28–60 equiv.

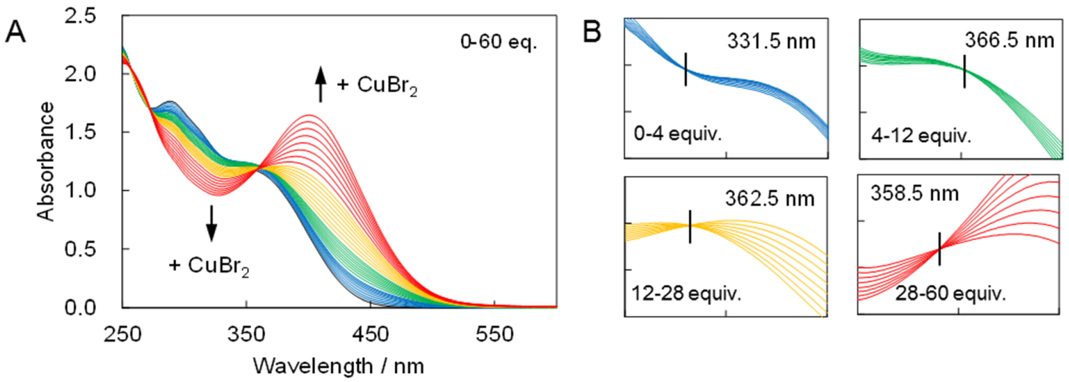

Supplementary Figure 9
Accumulation of Cu on TPM-G4. (A) Change in UV-vis absorption spectra of TPM-G4 in DCM/AN = 9:1 upon the addition of $Cu^{II}Br_2$ in AN up to 60 equiv. ([TPM-G4] = ca. 3.0×10^{-6} M, 20 °C). (B) Isosbestic points during complexation with $Cu^{II}Br_2$ in respective layers 0–4, 4–12, 12–28 and 28–60 equiv.

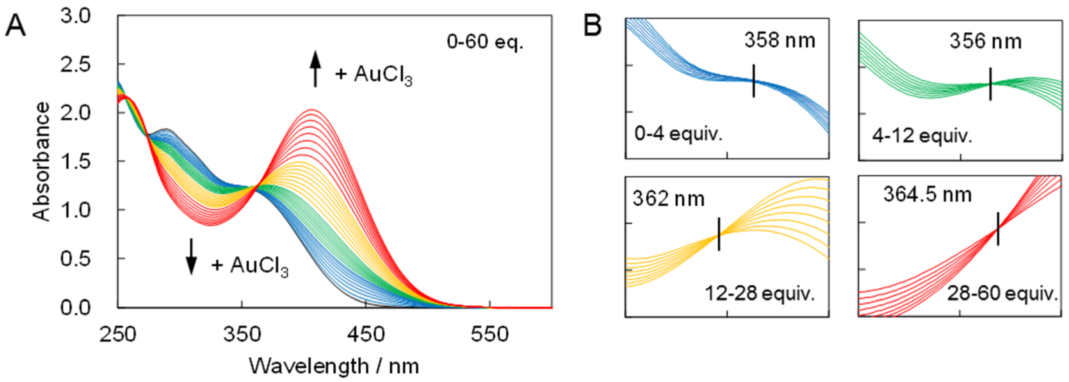

Supplementary Figure 10

Accumulation of Au on TPM-G4. (A) Change in UV-vis absorption spectra of TPM-G4 in DCM/AN = 2:1 upon the addition of $Au^{III}Cl_3$ in AN up to 60 equiv. ([TPM-G4] = ca. 3.0×10^{-6} M, 20 °C). (B) Isosbestic points during complexation with $Au^{III}Cl_3$ in respective layers 0–4, 4–12, 12–28 and 28–60 equiv.

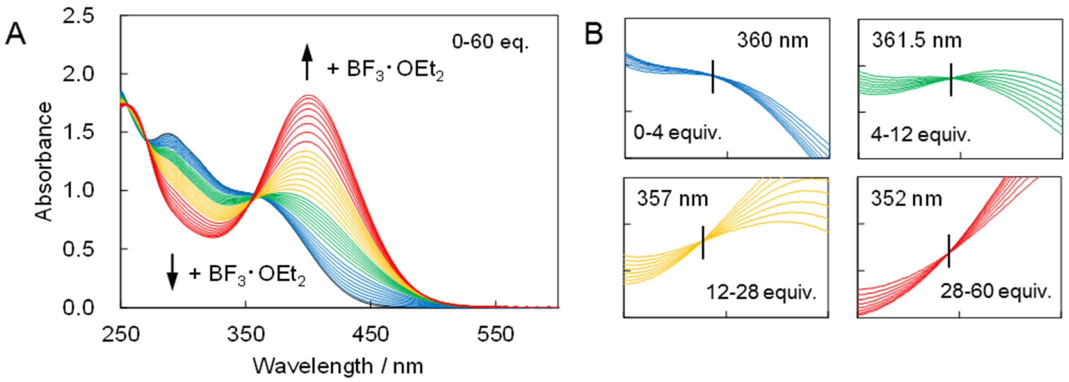

Supplementary Figure 11
Accumulation of B on TPM-G4. (A) Change in UV-vis absorption spectra of TPM-G4 in DOX/AN = 6:5 upon the addition of $B^{III}F_3(OEt_2)$ in AN up to 60 equiv. ([TPM-G4] = ca. 3.0×10^{-6} M, 20 °C, under N_2). (B) Isosbestic points during complexation with $B^{III}F_3(OEt_2)$ in respective layers 0–4, 4–12, 12–28 and 28–60 equiv.

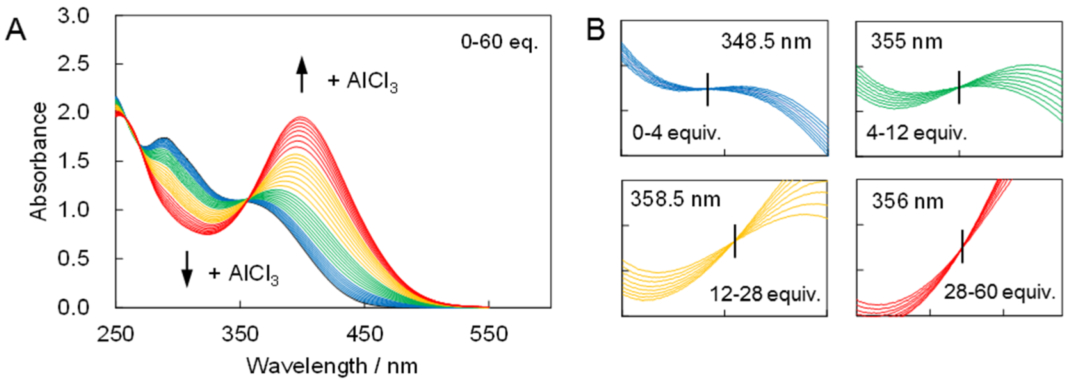

Supplementary Figure 12
Accumulation of Al on TPM-G4. (A) Change in UV-vis absorption spectra of TPM-G4 in DOX upon the addition of $Al^{III}Cl_3$ in DOX up to 60 equiv. ([TPM-G4] = ca. 3.0×10^{-6} M, 20 °C, under N_2). (B) Isosbestic points during complexation with $Al^{III}Cl_3$ in respective layers 0–4, 4–12, 12–28 and 28–60 equiv.

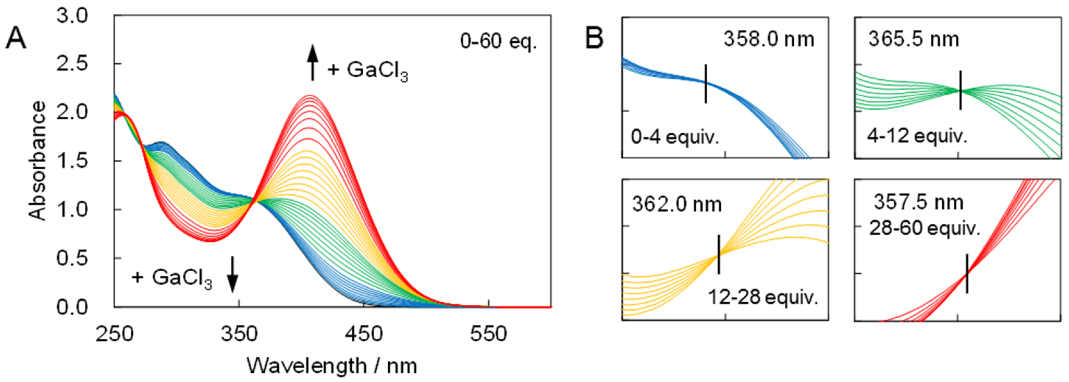

Supplementary Figure 13

Accumulation of Ga on TPM-G4. (A) Change in UV-vis absorption spectra of TPM-G4 in CLF/AN = 1:1 upon the addition of $Ga^{III}Cl_3$ in AN up to 60 equiv. ([TPM-G4] = ca. 3.0×10^{-6} M, 20 °C). (B) Isosbestic points during complexation with $Ga^{III}Cl_3$ in respective layers 0–4, 4–12, 12–28 and 28–60 equiv.

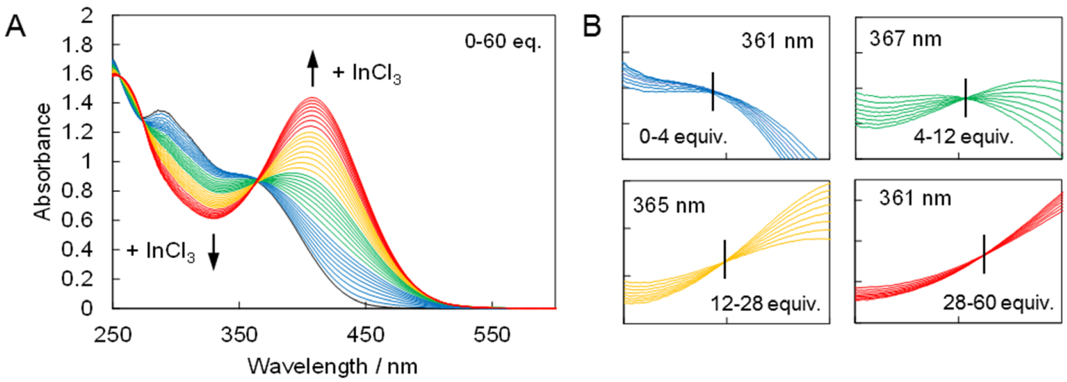

Supplementary Figure 14

Accumulation of In on TPM-G4. (A) Change in UV-vis absorption spectra of TPM-G4 in DCM/AN = 3:1 upon the addition of $In^{III}Cl_3$ in AN up to 60 equiv. ([TPM-G4] = ca. 3.0 × 10^{-6} M, 20 °C). (B) Isosbestic points during complexation with $In^{III}Cl_3$ in respective layers 0–4, 4–12, 12–28 and 28–60 equiv.

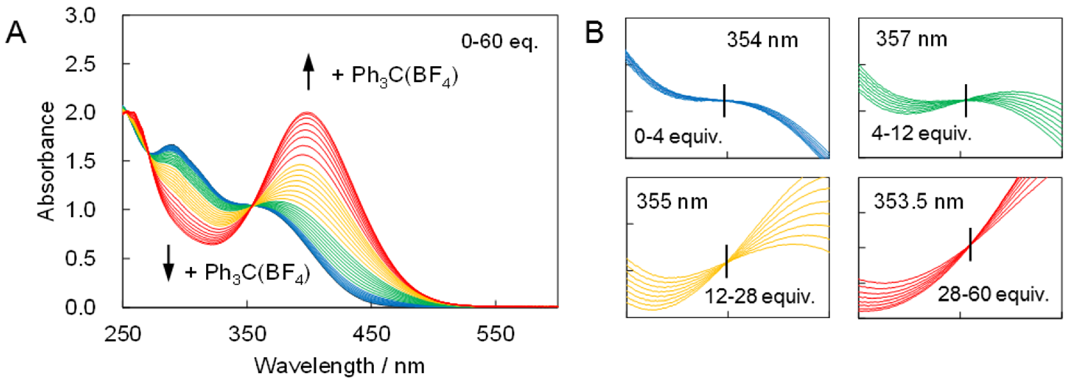

Supplementary Figure 15

Accumulation of C on TPM-G4. (A) Change in UV-vis absorption spectra of TPM-G4 in DOX/THF = 20:1 upon the addition of $[Ph_3C](BF_4)$ in AN up to 60 equiv. ([TPM-G4] = ca. 3.0×10^{-6} M, 20 °C, under N_2). (B) Isosbestic points during complexation with $[Ph_3C](BF_4)$ in respective layers 0–4, 4–12, 12–28 and 28–60 equiv.

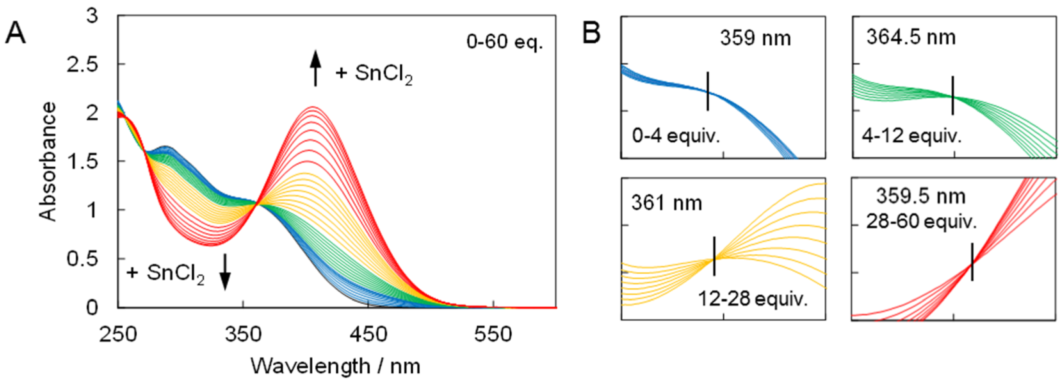

Supplementary Figure 16

Accumulation of Sn on TPM-G4. (A) Change in UV-vis absorption spectra of TPM-G4 in DCM/AN = 1:1 upon the addition of $Sn^{II}Cl_2$ in AN up to 60 equiv. ([TPM-G4] = ca. 3.0×10^{-6} M, 20 °C). (B) Isosbestic points during complexation with $Sn^{II}Cl_2$ in respective layers 0–4, 4–12, 12–28 and 28–60 equiv.

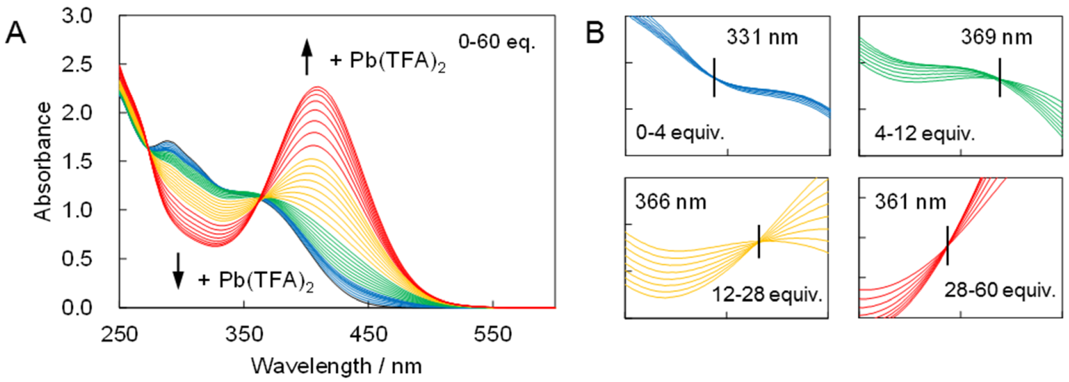

Supplementary Figure 17

Accumulation of Pb on TPM-G4. (A) Change in UV-vis absorption spectra of TPM-G4 in DCM upon the addition of $Pb^{II}(tfa)_2(aq)_{1/2}$ in DCM/AN = 9:1 up to 60 equiv. ([TPM-G4] = ca. 3.0×10^{-6} M, 20 °C). (B) Isosbestic points during complexation with $Pb^{II}(tfa)_2(aq)_{1/2}$ in respective layers 0–4, 4–12, 12–28 and 28–60 equiv.

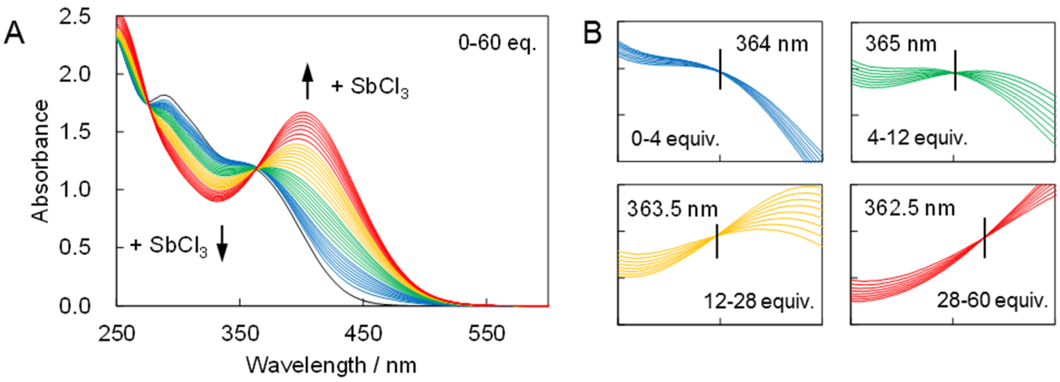

Supplementary Figure 18

Accumulation of Sb on TPM-G4. (A) Change in UV-vis absorption spectra of TPM-G4 in DCM/AN = 3:1 upon the addition of $Sb^{III}Cl_3$ in AN up to 60 equiv. ([TPM-G4] = ca. 3.0×10^{-6} M, 20 °C). (B) Isosbestic points during complexation with $Sb^{III}Cl_3$ in respective layers 0–4, 4–12, 12–28 and 28–60 equiv.

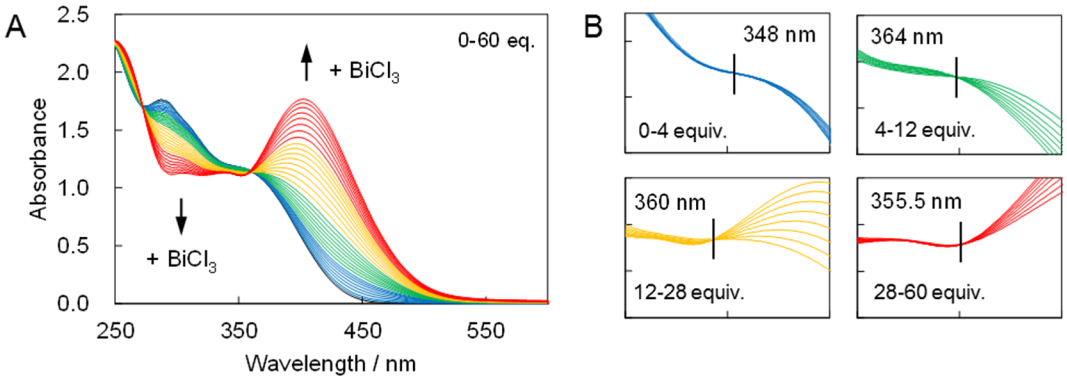

Supplementary Figure 19

Accumulation of Bi on TPM-G4. (A) Change in UV-vis absorption spectra of TPM-G4 in DCM/AN = 1:1 upon the addition of $Bi^{III}Cl_3$ in AN up to 60 equiv. ([TPM-G4] = ca. 3.0 × 10^{-6} M, 20 °C). (B) Isosbestic points during complexation with $Bi^{III}Cl_3$ in respective layers 0–4, 4–12, 12–28 and 28–60 equiv.

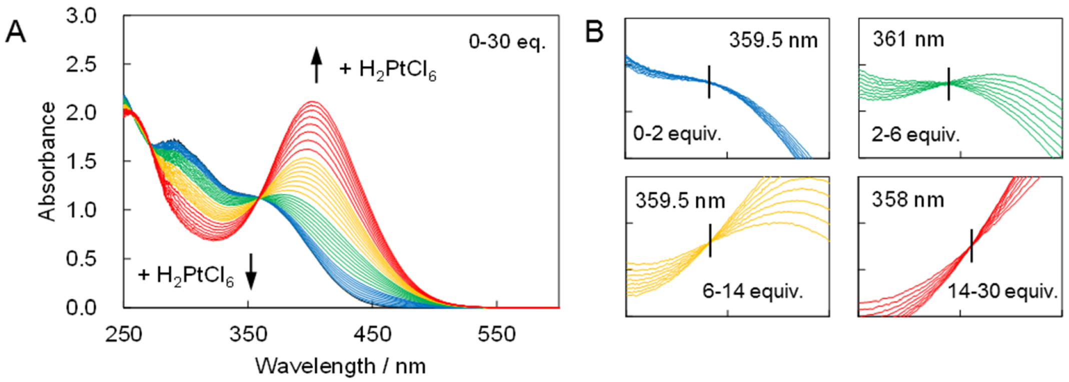

Supplementary Figure 20

Accumulation of H on TPM-G4. (A) Change in UV-vis absorption spectra of TPM-G4 in DOX/AN = 1:1 upon the addition of $H^{I}_2[PtCl_6]$ in AN up to 30 equiv. ([PyTPM-G4] = ca. 3.0×10^{-6} M, 20 °C). (B) Isosbestic points during complexation with $H^{I}_2[PtCl_6]$ in respective layers 0–2, 2–6, 6–14 and 14–30 equiv.

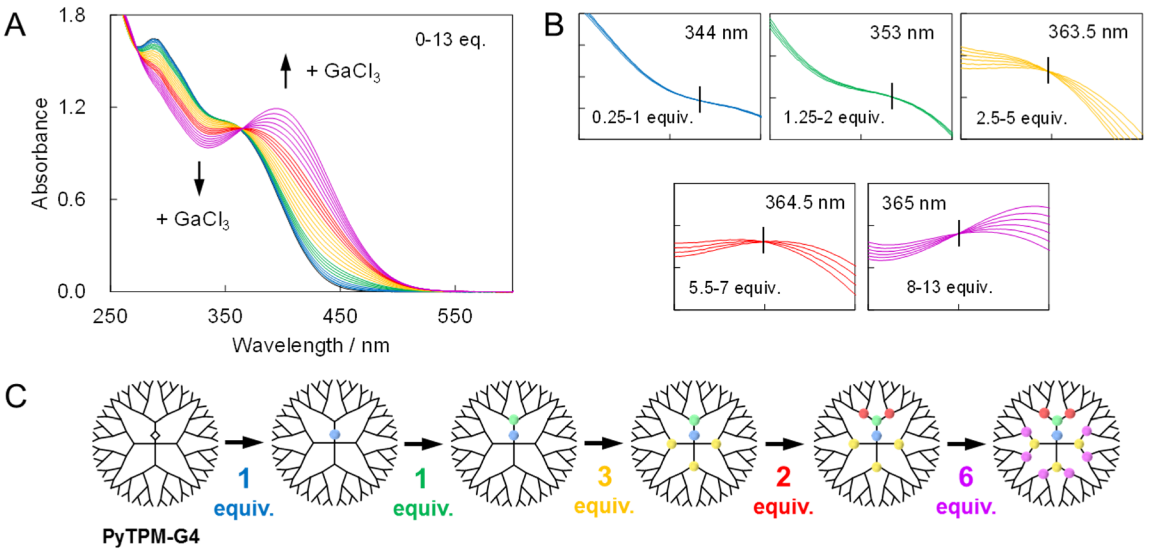

Supplementary Figure 21

Accumulation of Ga on PyTPM-G4. (A) Change in UV-vis absorption spectra of PyTPM-G4 upon the addition of $Ga^{III}Cl_3$ up to 13 equiv. in DCM/AN = 1:1 ([PyTPM-G4] = ca. 3.0×10^{-6} M, 20 °C). (B) Isosbestic points during complexation with $Ga^{III}Cl_3$ in respective layers 0–1, 1–2, 2–5, 5–7 and 7–13 equiv. (C) Layer-by-layer stepwise accumulation of a metal salt on PyTPM-G4.

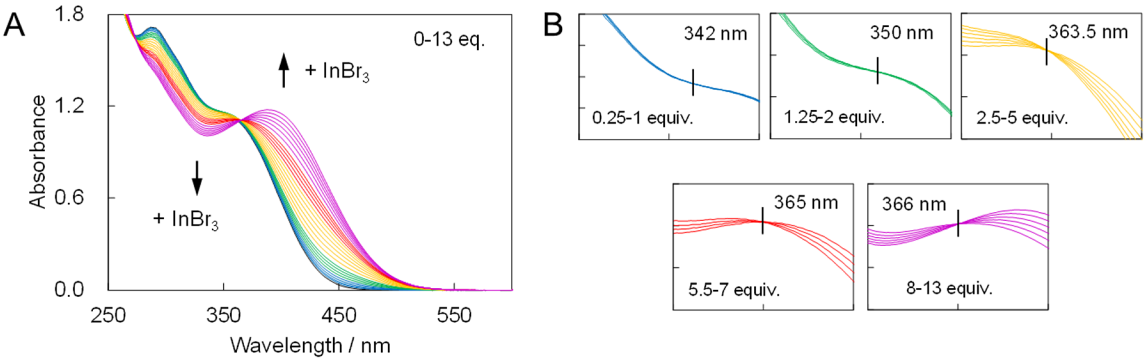

Supplementary Figure 22

Accumulation of In on PyTPM-G4. (A) Change in UV-vis absorption spectra of PyTPM-G4 upon the addition of $In^{III}Br_3$ up to 13 equiv. in DCM/AN = 1:1 ([PyTPM-G4] = ca. 3.0×10^{-6} M, 20 °C). (B) Isosbestic points during complexation with $In^{III}Br_3$ in respective layers 0–1, 1–2, 2–5, 5–7 and 7–13 equiv.

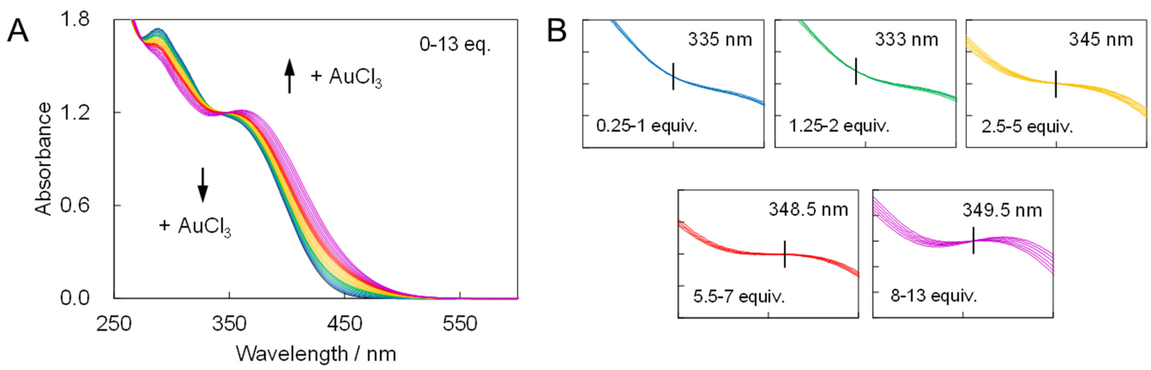

Supplementary Figure 23

Accumulation of Au on PyTPM-G4. (A) Change in UV-vis absorption spectra of PyTPM-G4 upon the addition of $Au^{III}Cl_3$ up to 13 equiv. in DCM/AN = 1:1 ([PyTPM-G4] = ca. 3.0×10^{-6} M, 20 °C). (B) Isosbestic points during complexation with $Au^{III}Cl_3$ in respective layers 0–1, 1–2, 2–5, 5–7 and 7–13 equiv.

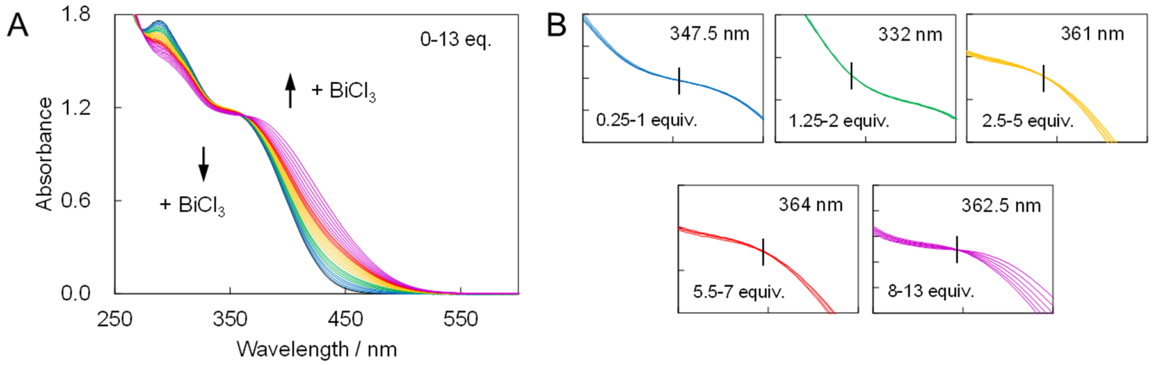

Supplementary Figure 24

Accumulation of Bi on PyTPM-G4. (A) Change in UV-vis absorption spectra of PyTPM-G4 upon the addition of $Bi^{III}Cl_3$ up to 13 equiv. in DCM/AN = 1:1 ([PyTPM-G4] = ca. 3.0×10^{-6} M, 20 °C). (B) Isosbestic points during complexation with $Bi^{III}Cl_3$ in respective layers 0–1, 1–2, 2–5, 5–7 and 7–13 equiv.

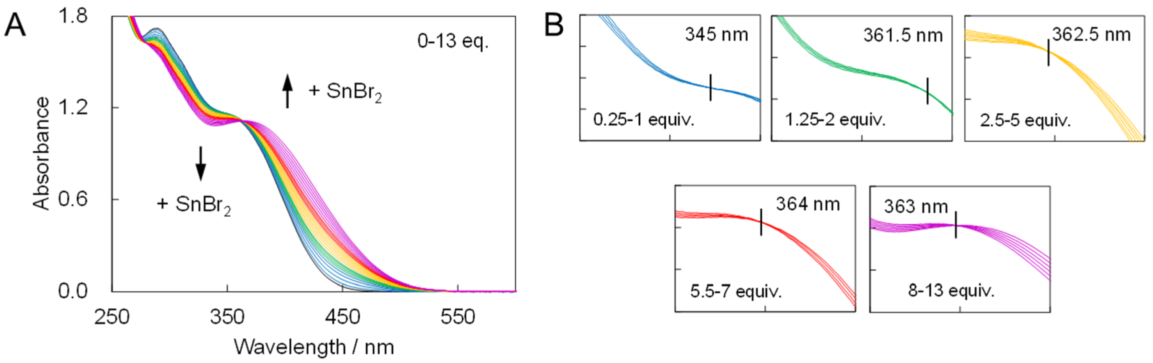

Supplementary Figure 25
Accumulation of Sn on PyTPM-G4. (A) Change in UV-vis absorption spectra of PyTPM-G4 upon the addition of $Sn^{II}Br_2$ up to 13 equiv. in DCM/AN = 1:1 ([PyTPM-G4] = ca. 3.0×10^{-6} M, 20 °C). (B) Isosbestic points during complexation with $Sn^{II}Br_2$ in respective layers 0–1, 1–2, 2–5, 5–7 and 7–13 equiv.

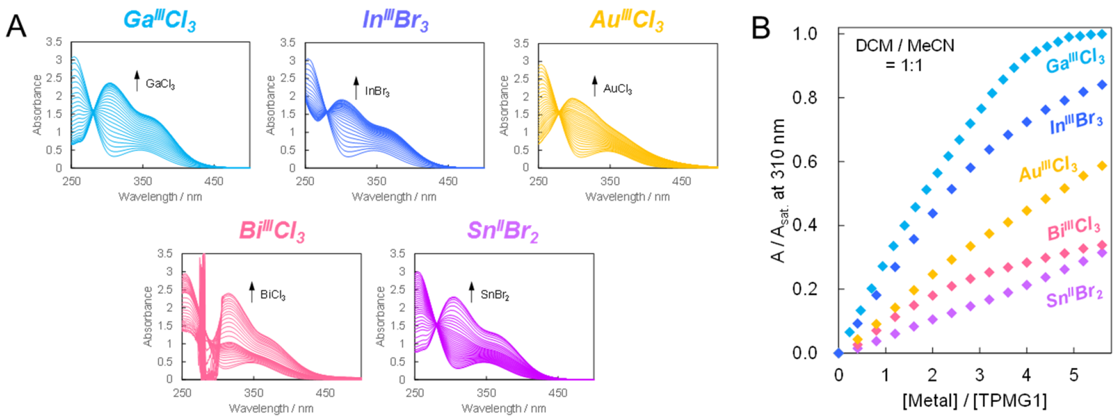

Supplementary Figure 26

Coordination strengths of $Ga^{III}Cl_3$, $In^{III}Br_3$, $Au^{III}Cl_3$, $Bi^{III}Cl_3$ and $Sn^{II}Br_2$ estimated by titration using TPM-G1. (A) Changes in UV-vis absorption spectra of TPM-G1 upon the addition of $Ga^{III}Cl_3$, $In^{III}Br_3$, $Au^{III}Cl_3$, $Bi^{III}Cl_3$ and $Sn^{II}Br_2$ in DCM/AN = 1:1 ([TPM-G1] = ca. 3.0×10^{-5} M, 20 °C) (B) Titration curves of TPM-G1. $A/A_{sat.}$ is complexation ratio represented as a normalized optical difference originated with the complexation during the titration.

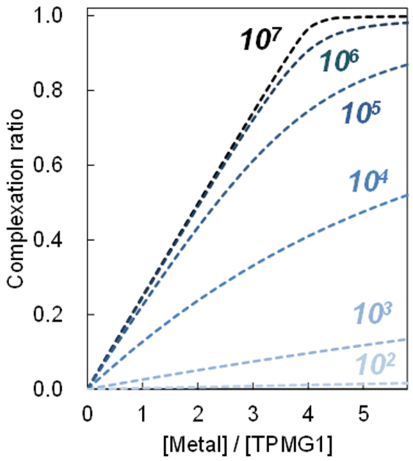

Supplementary Figure 27
Theoretical titration curves of TPM-G1 estimated from binding constants between metal salts and imine sites.

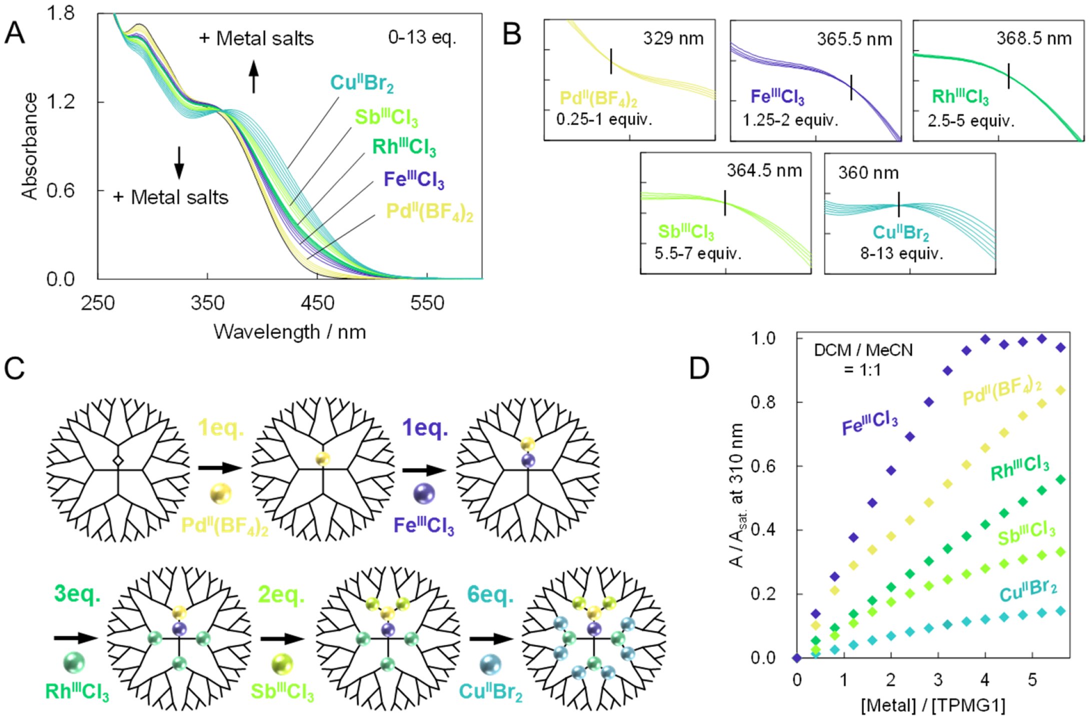

Supplementary Figure 28

Multimetallic accumulation of Fe, Pd, Rh, Sb and Cu on PyTPM-G4. (A) Changes in UV-vis absorption spectra of PyTPM-G4 upon the addition of 1 equiv. $Pd^{II}(BF_4)_2$, 1 equiv. $Fe^{III}Cl_3$, 3 equiv. $Rh^{III}Cl_3$, 2 equiv. $Sb^{III}Cl_3$ and 6 equiv. $Cu^{II}Br_2$ in DCM/AN = 1:1 ([PyTPM-G4] = ca. 3.0×10^{-6} M, 20 °C). Avoiding a reaction between Fe and Pd salts, $Pd^{II}(BF_4)_2$ was accumulated on PyTPM-G4 before the addition of $Fe^{III}Cl_3$. (B) Isosbestic points during complexation with the metal salts in respective layers 0–1, 1–2, 2–5, 5–7, 7–13 and 13–17. (C) Layer-by-layer stepwise multimetallic accumulation of the metal salts on PyTPM-G4. (D) Titration curves of TPM-G1 for $Fe^{III}Cl_3$, $Pd^{II}(BF_4)_2$, $Rh^{III}Cl_3$, $Sb^{III}Cl_3$ and $Cu^{II}Br_2$. $A/A_{sat.}$ is complexation ratio represented as a normalized optical difference originated with the complexation during the titration.

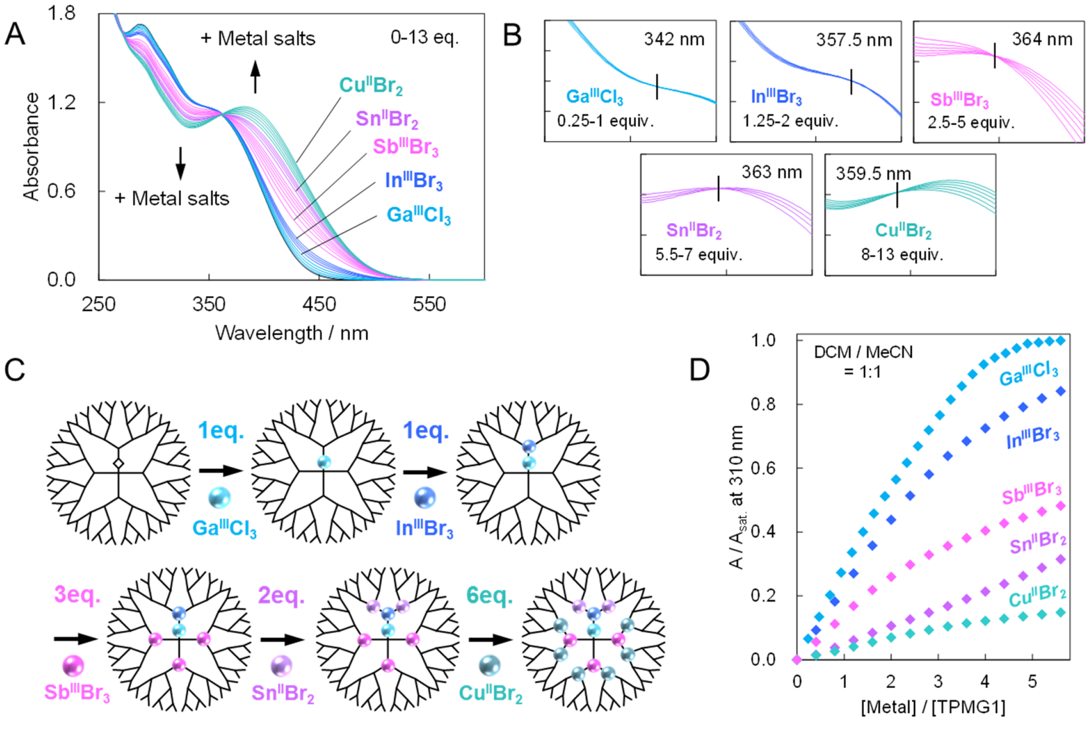

Supplementary Figure 29

Multimetallic accumulation of Ga, In, Sb, Sn and Cu on PyTPM-G4. (A) Changes in UV-vis absorption spectra of PyTPM-G4 upon the addition of 1 equiv. $Ga^{III}Cl_3$, 1 equiv. $In^{III}Br_3$, 3 equiv. $Sb^{III}Br_3$, 2 equiv. $Sn^{II}Br_2$ and 6 equiv. $Cu^{II}Br_2$ in DCM/AN = 1:1 ([PyTPM-G4] = ca. 3.0×10^{-6} M, 20 °C). (B) Isosbestic points during complexation with the metal salts in respective layers 0–1, 1–2, 2–5, 5–7, 7–13 and 13–17. (C) Layer-by-layer stepwise multimetallic accumulation of the metal salts on PyTPM-G4. (D) Titration curves of TPM-G1 for $Ga^{III}Cl_3$, $In^{III}Br_3$, $Sb^{III}Br_3$, $Sn^{II}Br_2$ and $Cu^{II}Br_2$. $A/A_{sat.}$ is complexation ratio represented as a normalized optical difference originated with the complexation during the titration.

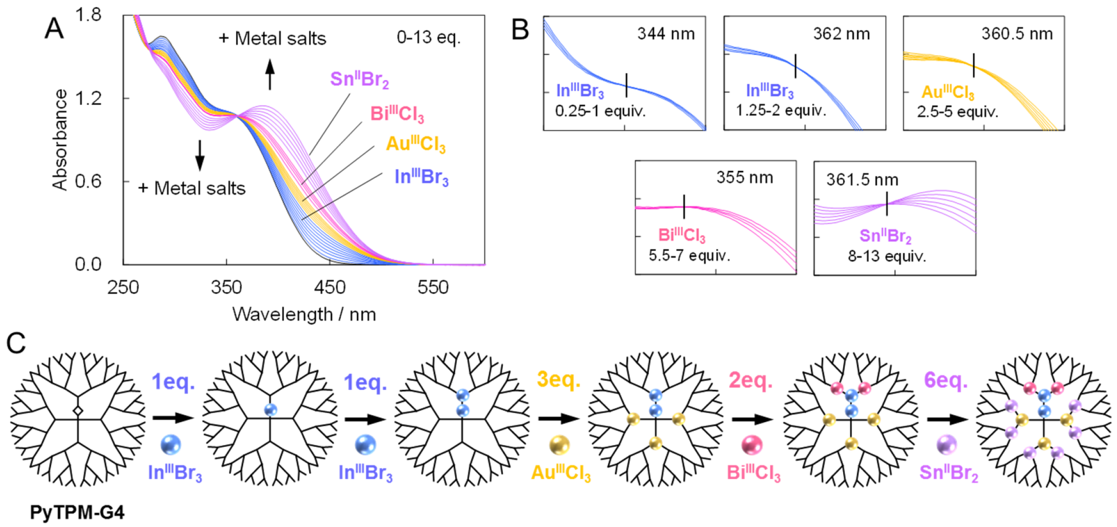

Supplementary Figure 30

Multimetallic accumulation of In, Au, Bi and Sn on PyTPM-G4. (A) Changes in UV-vis absorption spectra of PyTPM-G4 upon the addition of 2 equiv. $In^{III}Br_3$, 3 equiv. $Au^{III}Cl_3$, 2 equiv. $Bi^{III}Cl_3$ and 6 equiv. $Sn^{II}Br_2$ in DCM/AN = 1:1 ([PyTPM-G4] = ca. 3.0×10^{-6} M, 20 °C). (B) Isosbestic points during complexation with the metal salts in respective layers 0–1, 1–2, 2–5, 5–7 and 7–13. (C) Layer-by-layer stepwise multimetallic accumulation of the metal salts on PyTPM-G4.

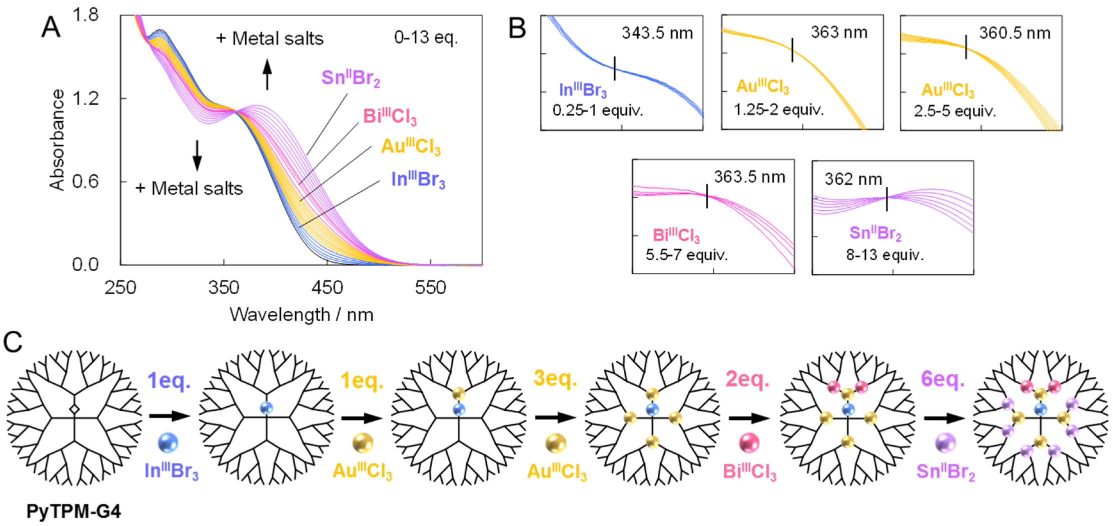

Supplementary Figure 31

Multimetallic accumulation of In, Au, Bi and Sn on PyTPM-G4. (A) Changes in UV-vis absorption spectra of PyTPM-G4 upon the addition of 1 equiv. $In^{III}Br_3$, 4 equiv. $Au^{III}Cl_3$, 2 equiv. $Bi^{III}Cl_3$ and 6 equiv. $Sn^{II}Br_2$ in DCM/AN = 1:1 ([PyTPM-G4] = ca. 3.0×10^{-6} M, 20 °C). (B) Isosbestic points during complexation with the metal salts in respective layers 0–1, 1–2, 2–5, 5–7 and 7–13. (C) Layer-by-layer stepwise multimetallic accumulation of the metal salts on PyTPM-G4.

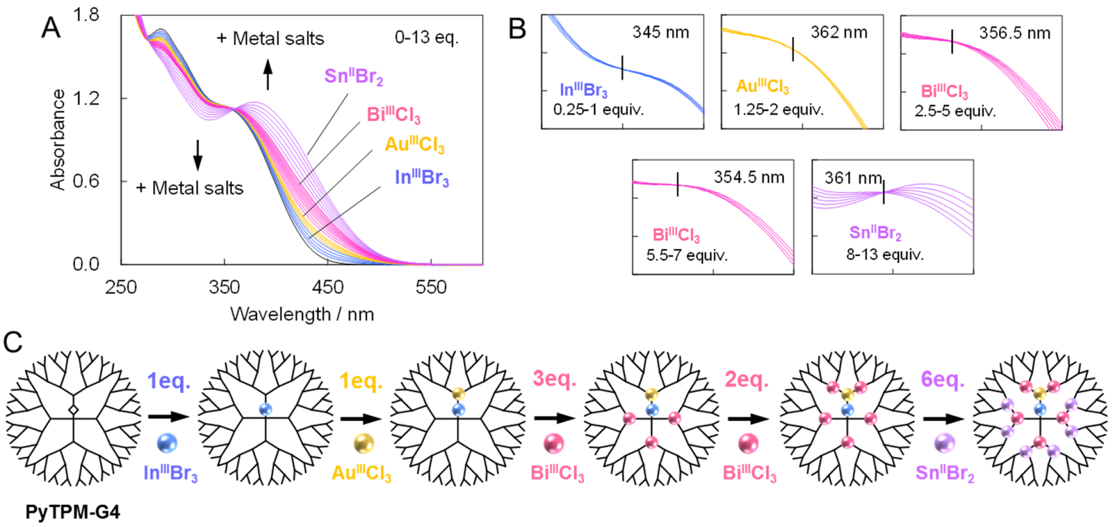

Supplementary Figure 32

Multimetallic accumulation of In, Au, Bi and Sn on PyTPM-G4. (A) Changes in UV-vis absorption spectra of PyTPM-G4 upon the addition of 1 equiv. $In^{III}Br_3$, 1 equiv. $Au^{III}Cl_3$, 5 equiv. $Bi^{III}Cl_3$ and 6 equiv. $Sn^{II}Br_2$ in DCM/AN = 1:1 ([PyTPM-G4] = ca. 3.0×10^{-6} M, 20 °C). (B) Isosbestic points during complexation with the metal salts in respective layers 0–1, 1–2, 2–5, 5–7 and 7–13. (C) Layer-by-layer stepwise multimetallic accumulation of the metal salts on PyTPM-G4.

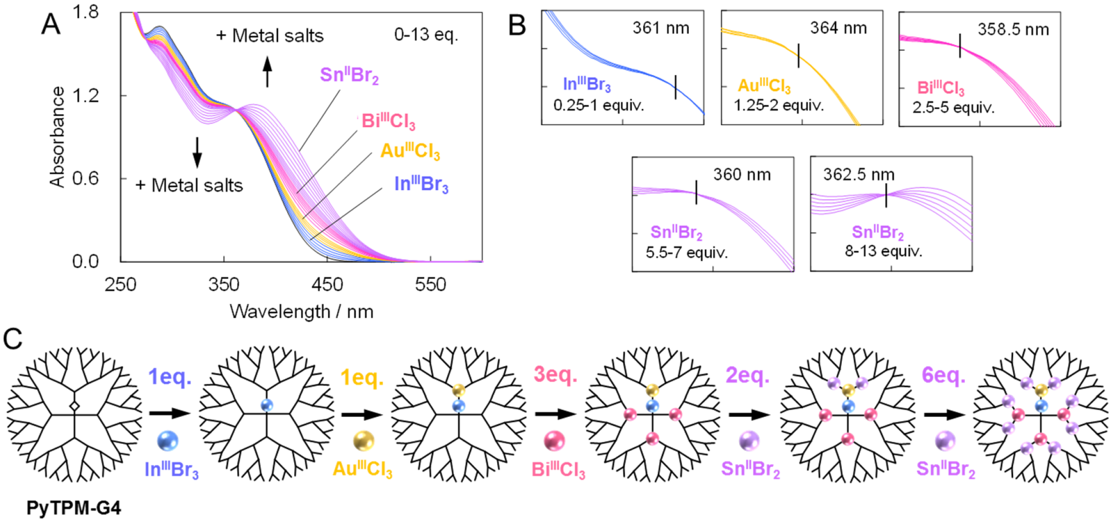

Supplementary Figure 33

Multimetallic accumulation of In, Au, Bi and Sn on PyTPM-G4. (A) Changes in UV-vis absorption spectra of PyTPM-G4 upon the addition of 1 equiv. $In^{III}Br_3$, 1 equiv. $Au^{III}Cl_3$, 3 equiv. $Bi^{III}Cl_3$ and 8 equiv. $Sn^{II}Br_2$ in DCM/AN = 1:1 ([PyTPM-G4] = ca. 3.0×10^{-6} M, 20 °C). (B) Isosbestic points during complexation with the metal salts in respective layers 0–1, 1–2, 2–5, 5–7 and 7–13. (C) Layer-by-layer stepwise multimetallic accumulation of the metal salts on PyTPM-G4.

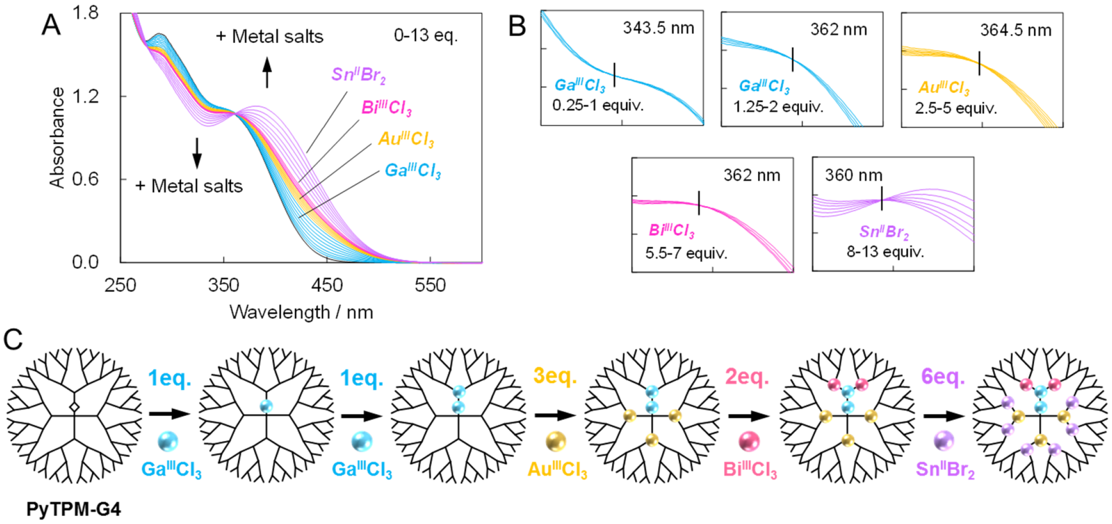

Supplementary Figure 34

Multimetallic accumulation of Ga, Au, Bi and Sn on PyTPM-G4. (A) Changes in UV-vis absorption spectra of PyTPM-G4 upon the addition of 2 equiv. $Ga^{III}Cl_3$, 3 equiv. $Au^{III}Cl_3$, 2 equiv. $Bi^{III}Cl_3$ and 6 equiv. $Sn^{II}Br_2$ in DCM/AN = 1:1 ([PyTPM-G4] = ca. 3.0×10^{-6} M, 20 °C). (B) Isosbestic points during complexation with the metal salts in respective layers 0–1, 1–2, 2–5, 5–7 and 7–13. (C) Layer-by-layer stepwise multimetallic accumulation of the metal salts on PyTPM-G4.

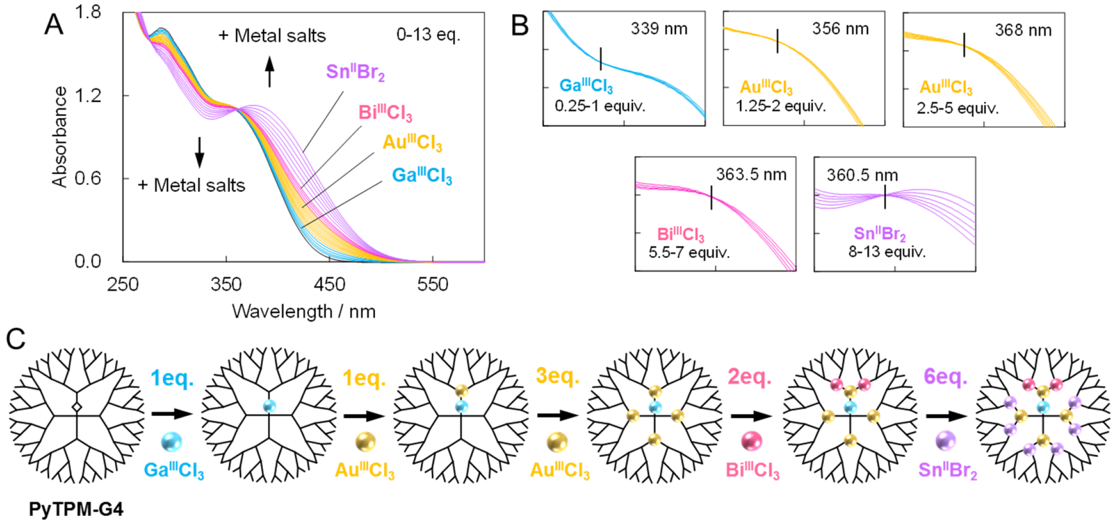

Supplementary Figure 35

Multimetallic accumulation of Ga, Au, Bi and Sn on PyTPM-G4. (A) Changes in UV-vis absorption spectra of PyTPM-G4 upon the addition of 1 equiv. $Ga^{III}Cl_3$, 4 equiv. $Au^{III}Cl_3$, 2 equiv. $Bi^{III}Cl_3$ and 6 equiv. $Sn^{II}Br_2$ in DCM/AN = 1:1 ([PyTPM-G4] = ca. 3.0×10^{-6} M, 20 °C). (B) Isosbestic points during complexation with the metal salts in respective layers 0–1, 1–2, 2–5, 5–7 and 7–13. (C) Layer-by-layer stepwise multimetallic accumulation of the metal salts on PyTPM-G4.

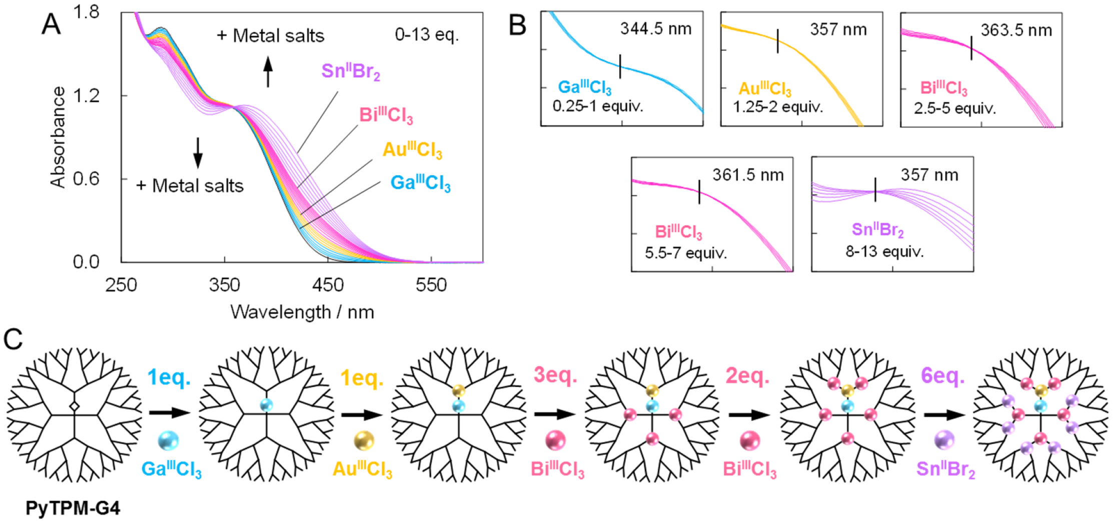

Supplementary Figure 36

Multimetallic accumulation of Ga, Au, Bi and Sn on PyTPM-G4. (A) Changes in UV-vis absorption spectra of PyTPM-G4 upon the addition of 1 equiv. $Ga^{III}Cl_3$, 1 equiv. $Au^{III}Cl_3$, 5 equiv. $Bi^{III}Cl_3$ and 6 equiv. $Sn^{II}Br_2$ in DCM/AN = 1:1 ([PyTPM-G4] = ca. 3.0×10^{-6} M, 20 °C). (B) Isosbestic points during complexation with the metal salts in respective layers 0–1, 1–2, 2–5, 5–7 and 7–13. (C) Layer-by-layer stepwise multimetallic accumulation of the metal salts on PyTPM-G4.

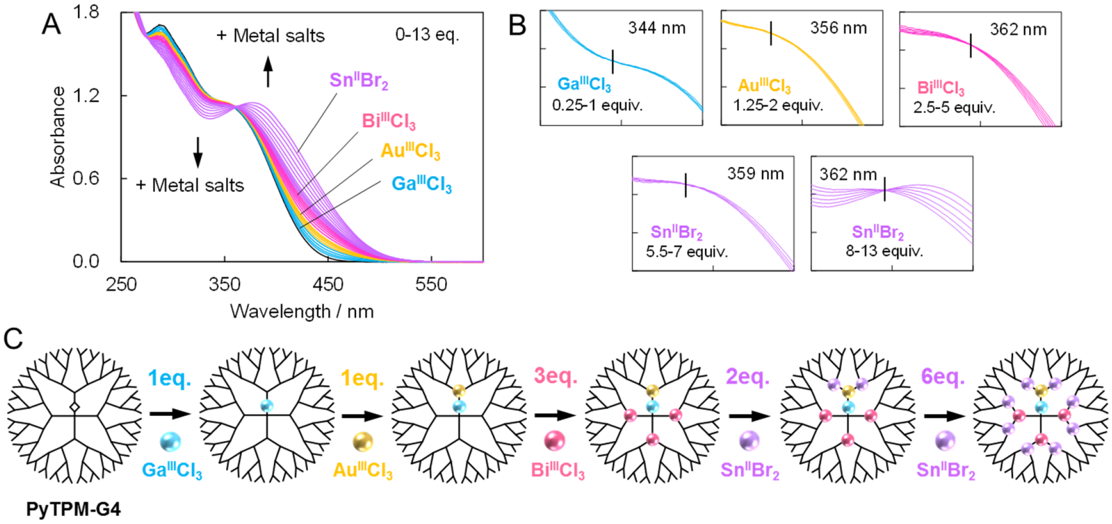

Supplementary Figure 37

Multimetallic accumulation of Ga, Au, Bi and Sn on PyTPM-G4. (A) Changes in UV-vis absorption spectra of PyTPM-G4 upon the addition of 1 equiv. $Ga^{III}Cl_3$, 1 equiv. $Au^{III}Cl_3$, 3 equiv. $Bi^{III}Cl_3$ and 8 equiv. $Sn^{II}Br_2$ in DCM/AN = 1:1 ([PyTPM-G4] = ca. 3.0×10^{-6} M, 20 °C). (B) Isosbestic points during complexation with the metal salts in respective layers 0–1, 1–2, 2–5, 5–7 and 7–13. (C) Layer-by-layer stepwise multimetallic accumulation of the metal salts on PyTPM-G4.

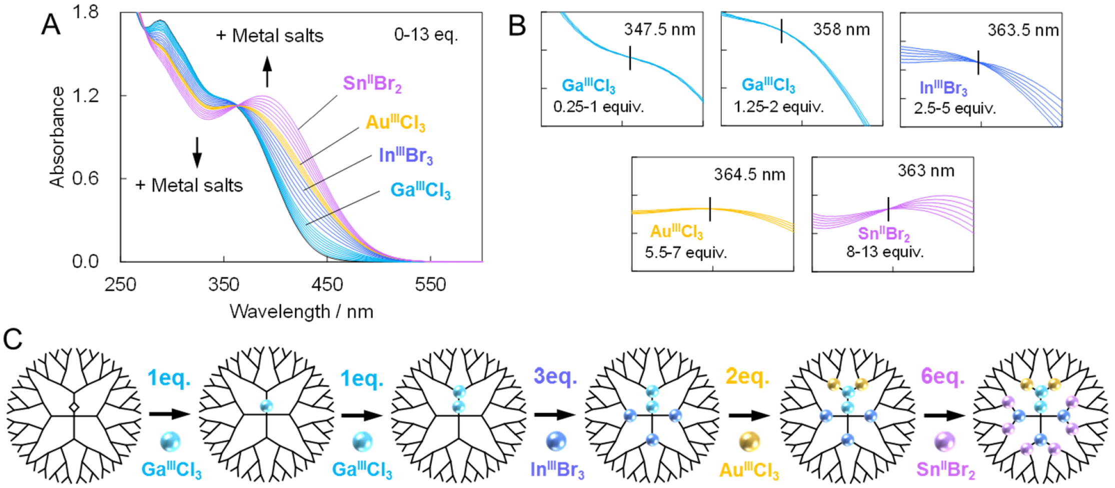

Supplementary Figure 38

Multimetallic accumulation of Ga, In, Au and Sn on PyTPM-G4. (A) Changes in UV-vis absorption spectra of PyTPM-G4 upon the addition of 2 equiv. $Ga^{III}Cl_3$, 3 equiv. $In^{III}Br_3$, 2 equiv. $Au^{III}Cl_3$ and 6 equiv. $Sn^{II}Br_2$ in DCM/AN = 1:1 ([PyTPM-G4] = ca. 3.0×10^{-6} M, 20 °C). (B) Isosbestic points during complexation with the metal salts in respective layers 0–1, 1–2, 2–5, 5–7 and 7–13. (C) Layer-by-layer stepwise multimetallic accumulation of the metal salts on PyTPM-G4.

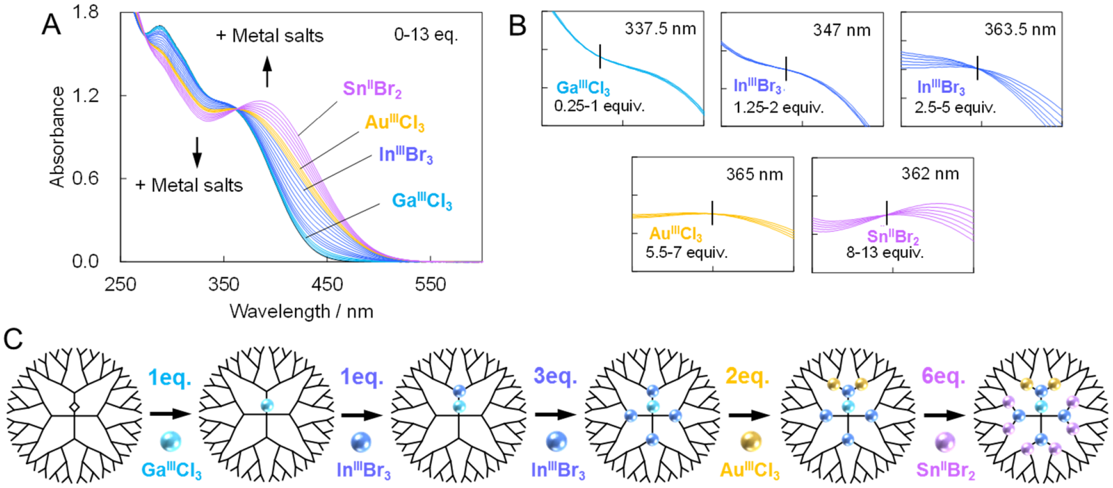

Supplementary Figure 39

Multimetallic accumulation of Ga, In, Au and Sn on PyTPM-G4. (A) Changes in UV-vis absorption spectra of PyTPM-G4 upon the addition of 1 equiv. $Ga^{III}Cl_3$, 4 equiv. $In^{III}Br_3$, 2 equiv. $Au^{III}Cl_3$ and 6 equiv. $Sn^{II}Br_2$ in DCM/AN = 1:1 ([PyTPM-G4] = ca. 3.0×10^{-6} M, 20 °C). (B) Isosbestic points during complexation with the metal salts in respective layers 0–1, 1–2, 2–5, 5–7 and 7–13. (C) Layer-by-layer stepwise multimetallic accumulation of the metal salts on PyTPM-G4.

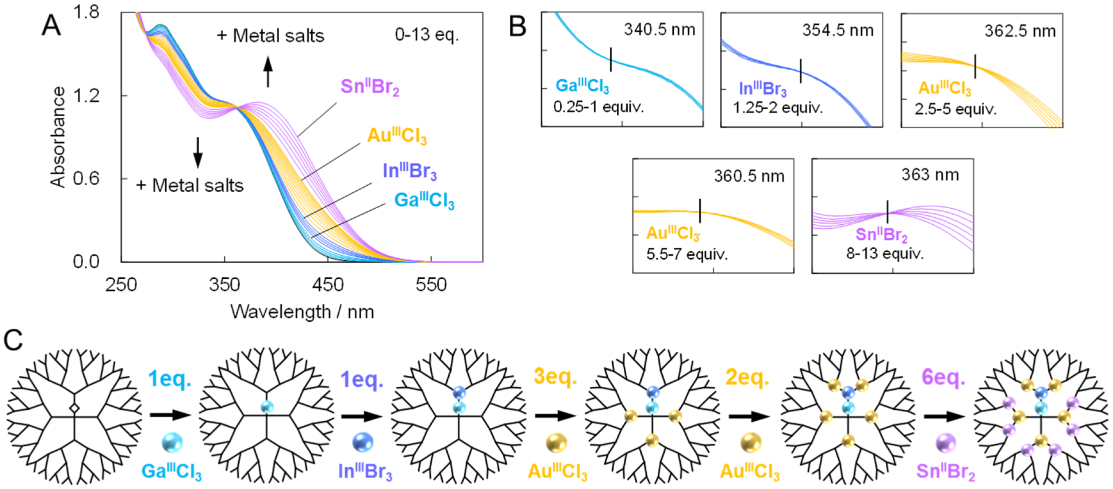

Supplementary Figure 40

Multimetallic accumulation of Ga, In, Au and Sn on PyTPM-G4. (A) Changes in UV-vis absorption spectra of PyTPM-G4 upon the addition of 1 equiv. $Ga^{III}Cl_3$, 1 equiv. $In^{III}Br_3$, 5 equiv. $Au^{III}Cl_3$ and 6 equiv. $Sn^{II}Br_2$ in DCM/AN = 1:1 ([PyTPM-G4] = ca. 3.0×10^{-6} M, 20 °C). (B) Isosbestic points during complexation with the metal salts in respective layers 0–1, 1–2, 2–5, 5–7 and 7–13. (C) Layer-by-layer stepwise multimetallic accumulation of the metal salts on PyTPM-G4.

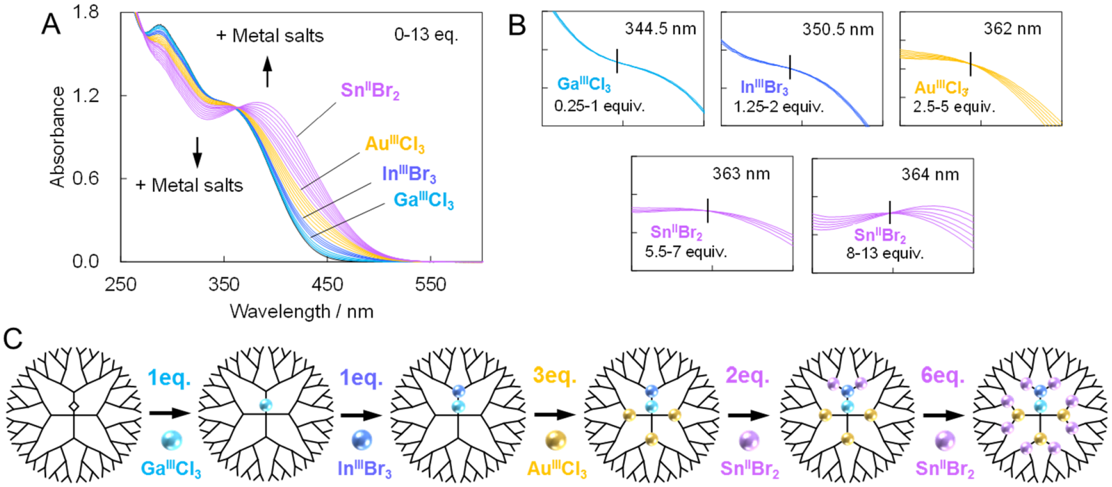

Supplementary Figure 41

Multimetallic accumulation of Ga, In, Au and Sn on PyTPM-G4. (A) Changes in UV-vis absorption spectra of PyTPM-G4 upon the addition of 1 equiv. $Ga^{III}Cl_3$, 1 equiv. $In^{III}Br_3$, 3 equiv. $Au^{III}Cl_3$ and 8 equiv. $Sn^{II}Br_2$ in DCM/AN = 1:1 ([PyTPM-G4] = ca. 3.0×10^{-6} M, 20 °C). (B) Isosbestic points during complexation with the metal salts in respective layers 0–1, 1–2, 2–5, 5–7 and 7–13. (C) Layer-by-layer stepwise multimetallic accumulation of the metal salts on PyTPM-G4.

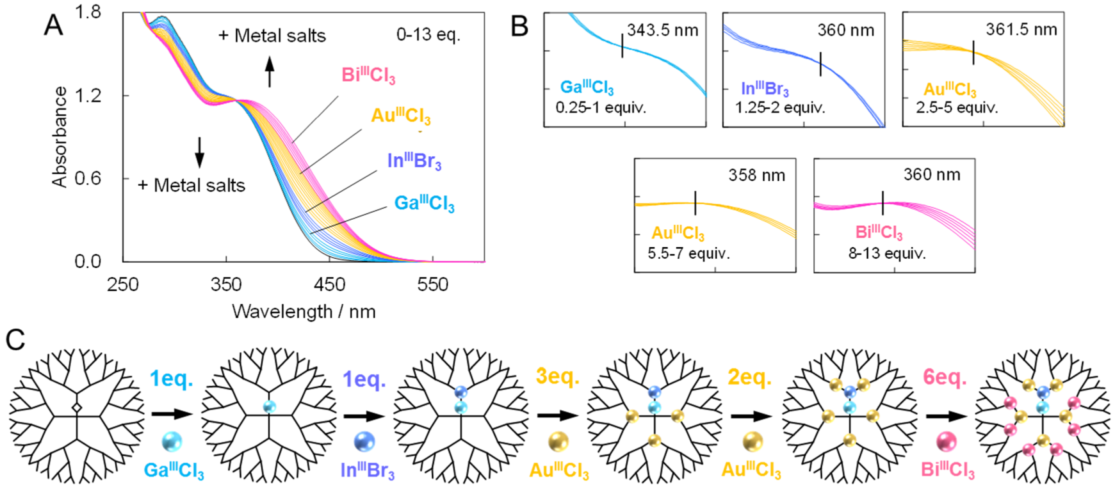

Supplementary Figure 42

Multimetallic accumulation of Ga, In, Au and Bi on PyTPM-G4. (A) Changes in UV-vis absorption spectra of PyTPM-G4 upon the addition of 1 equiv. $Ga^{III}Cl_3$, 1 equiv. $In^{III}Br_3$, 5 equiv. $Au^{III}Cl_3$ and 6 equiv. $Bi^{III}Cl_3$ in DCM/AN = 1:1 ([PyTPM-G4] = ca. 3.0×10^{-6} M, 20 °C). (B) Isosbestic points during complexation with the metal salts in respective layers 0–1, 1–2, 2–5, 5–7 and 7–13. (C) Layer-by-layer stepwise multimetallic accumulation of the metal salts on PyTPM-G4.

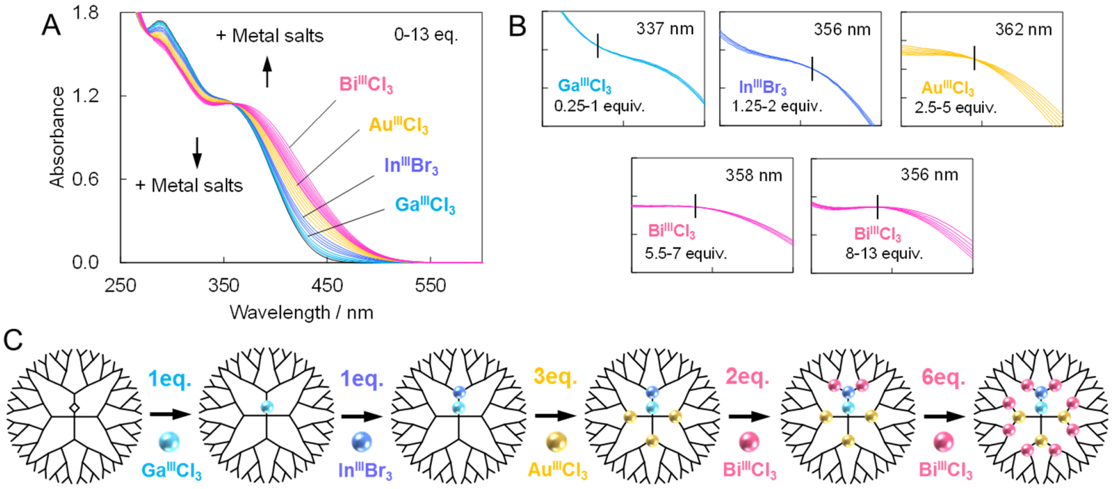

Supplementary Figure 43

Multimetallic accumulation of Ga, In, Au and Bi on PyTPM-G4. (A) Changes in UV-vis absorption spectra of PyTPM-G4 upon the addition of 1 equiv. $Ga^{III}Cl_3$, 1 equiv. $In^{III}Br_3$, 3 equiv. $Au^{III}Cl_3$ and 8 equiv. $Bi^{III}Cl_3$ in DCM/AN = 1:1 ([PyTPM-G4] = ca. 3.0×10^{-6} M, 20 °C). (B) Isosbestic points during complexation with the metal salts in respective layers 0–1, 1–2, 2–5, 5–7 and 7–13. (C) Layer-by-layer stepwise multimetallic accumulation of the metal salts on PyTPM-G4.

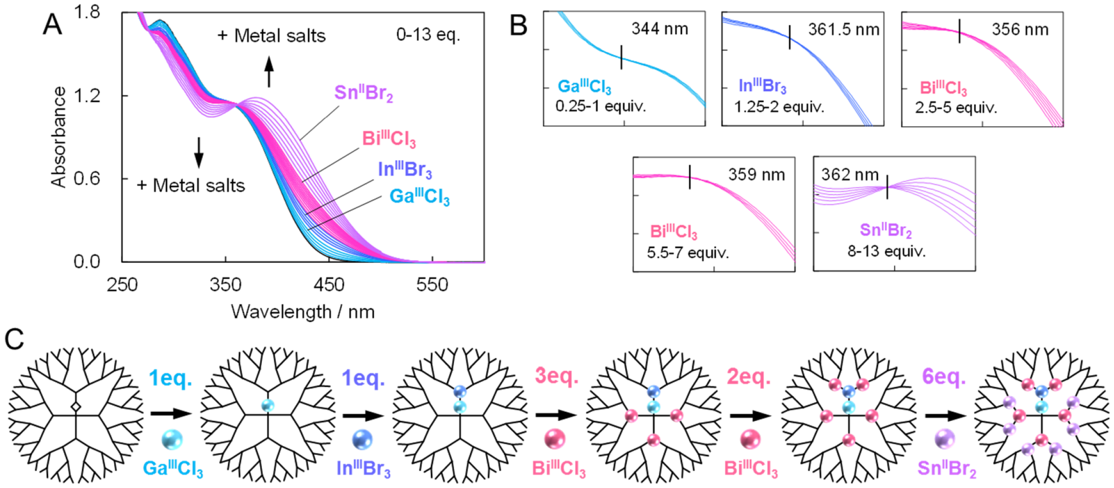

Supplementary Figure 44

Multimetallic accumulation of Ga, In, Bi and Sn on PyTPM-G4. (A) Changes in UV-vis absorption spectra of PyTPM-G4 upon the addition of 1 equiv. $Ga^{III}Cl_3$, 1 equiv. $In^{III}Br_3$, 5 equiv. $Bi^{III}Cl_3$ and 6 equiv. $Sn^{II}Br_2$ in DCM/AN = 1:1 ([PyTPM-G4] = ca. 3.0×10^{-6} M, 20 °C). (B) Isosbestic points during complexation with the metal salts in respective layers 0–1, 1–2, 2–5, 5–7 and 7–13. (C) Layer-by-layer stepwise multimetallic accumulation of the metal salts on PyTPM-G4.

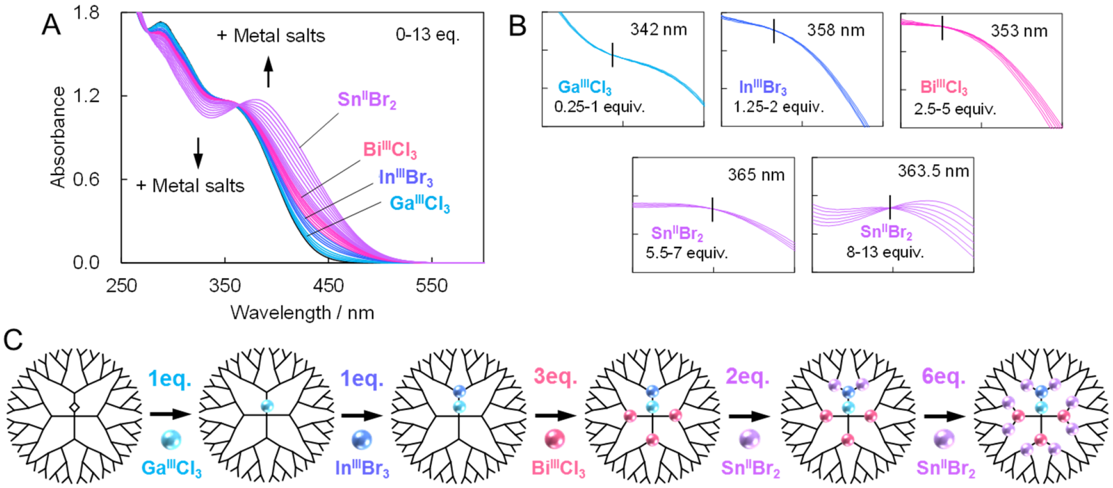

Supplementary Figure 45

Multimetallic accumulation of Ga, In, Bi and Sn on PyTPM-G4. (A) Changes in UV-vis absorption spectra of PyTPM-G4 upon the addition of 1 equiv. $Ga^{III}Cl_3$, 1 equiv. $In^{III}Br_3$, 3 equiv. $Bi^{III}Cl_3$ and 8 equiv. $Sn^{II}Br_2$ in DCM/AN = 1:1 ([PyTPM-G4] = ca. 3.0×10^{-6} M, 20 °C). (B) Isosbestic points during complexation with the metal salts in respective layers 0–1, 1–2, 2–5, 5–7 and 7–13. (C) Layer-by-layer stepwise multimetallic accumulation of the metal salts on PyTPM-G4.

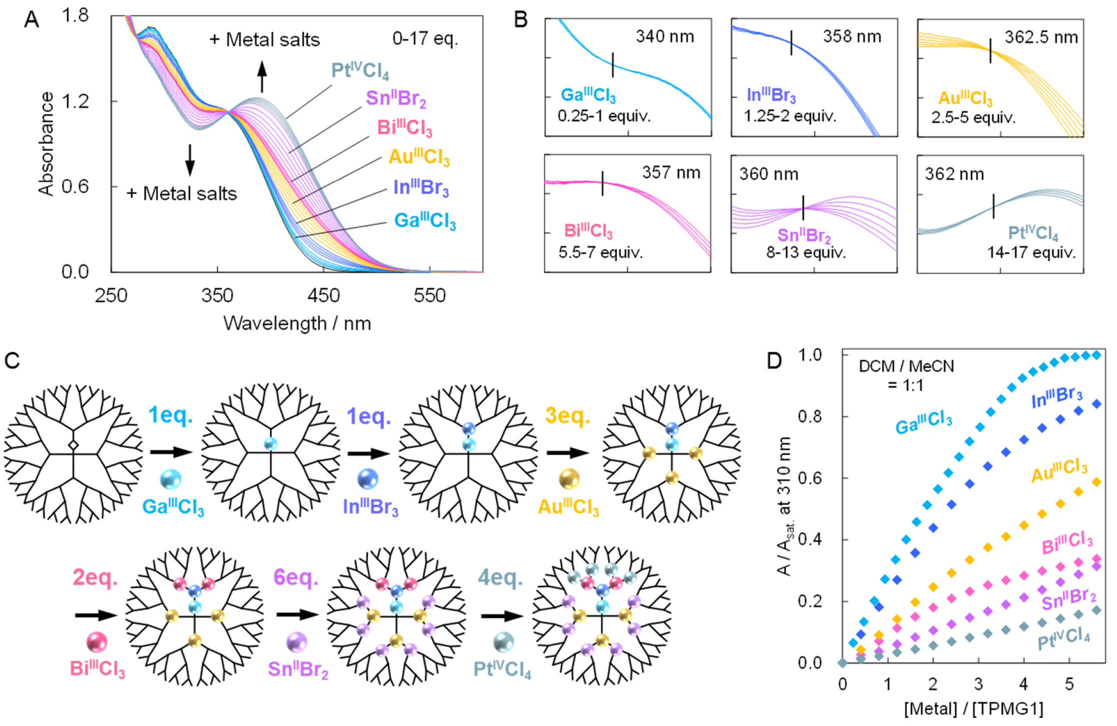

Supplementary Figure 46

Multimetallic accumulation of Ga, In, Au, Bi, Sn and Pt on PyTPM-G4. (A) Changes in UV-vis absorption spectra of PyTPM-G4 upon the addition of 1 equiv. $Ga^{III}Cl_3$, 1 equiv. $In^{III}Br_3$, 3 equiv. $Au^{III}Cl_3$, 2 equiv. $Bi^{III}Cl_3$, 6 equiv. $Sn^{II}Br_2$ and 4 equiv. $Pt^{IV}Cl_4$ in DCM/AN = 1:1 ([PyTPM-G4] = ca. 3.0×10^{-6} M, 20 °C). (B) Isosbestic points during complexation with the metal salts in respective layers 0–1, 1–2, 2–5, 5–7, 7–13 and 13–17. (C) Layer-by-layer stepwise multimetallic accumulation of the metal salts on PyTPM-G4. (D) Titration curves of TPM-G1 for $Ga^{III}Cl_3$, $In^{III}Br_3$, $Au^{III}Cl_3$, $Bi^{III}Cl_3$, $Sn^{II}Br_2$ and $Pt^{IV}Cl_4$. $A/A_{sat.}$ is complexation ratio represented as a normalized optical difference originated with the complexation during the titration.

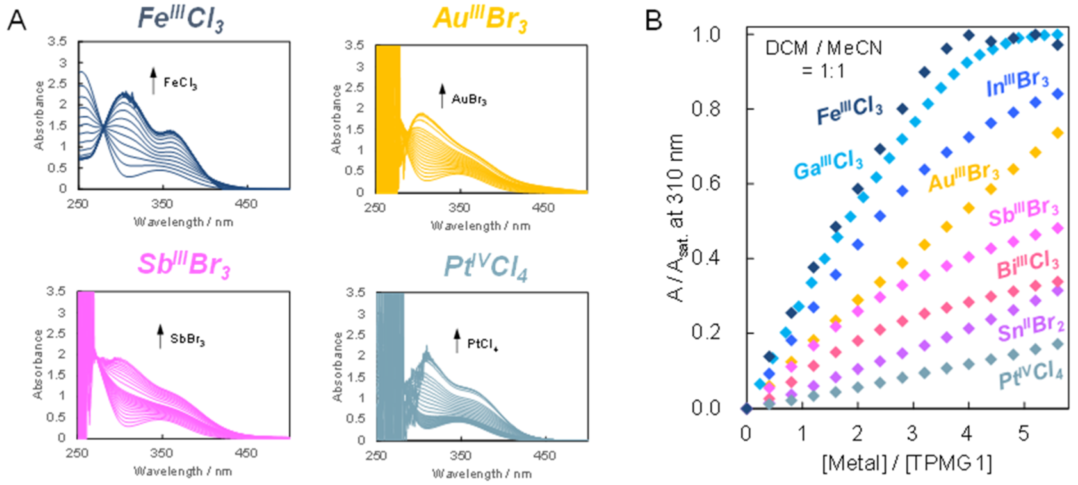

Supplementary Figure 47

Coordination strengths of $Fe^{III}Cl_3$, $Au^{III}Br_3$, $Sb^{III}Br_3$ and $Pt^{IV}Cl_4$ estimated by titration using TPM-G1. (A) Changes in UV-vis absorption spectra of TPM-G1 upon the addition of $Fe^{III}Cl_3$, $Au^{III}Br_3$, $Sb^{III}Br_3$ and $Pt^{IV}Cl_4$ in DCM/AN = 1:1 ([TPM-G1] = ca. 3.0×10^{-5} M, 20 °C) (B) Titration curves of TPM-G1 for $Fe^{III}Cl_3$, $Ga^{III}Cl_3$, $In^{III}Br_3$, $Au^{III}Br_3$, $Sb^{III}Br_3$, $Bi^{III}Cl_3$, $Sn^{II}Br_2$ and $Pt^{IV}Cl_4$. $A/A_{sat.}$ is complexation ratio represented as a normalized optical difference originated with the complexation during the titration.

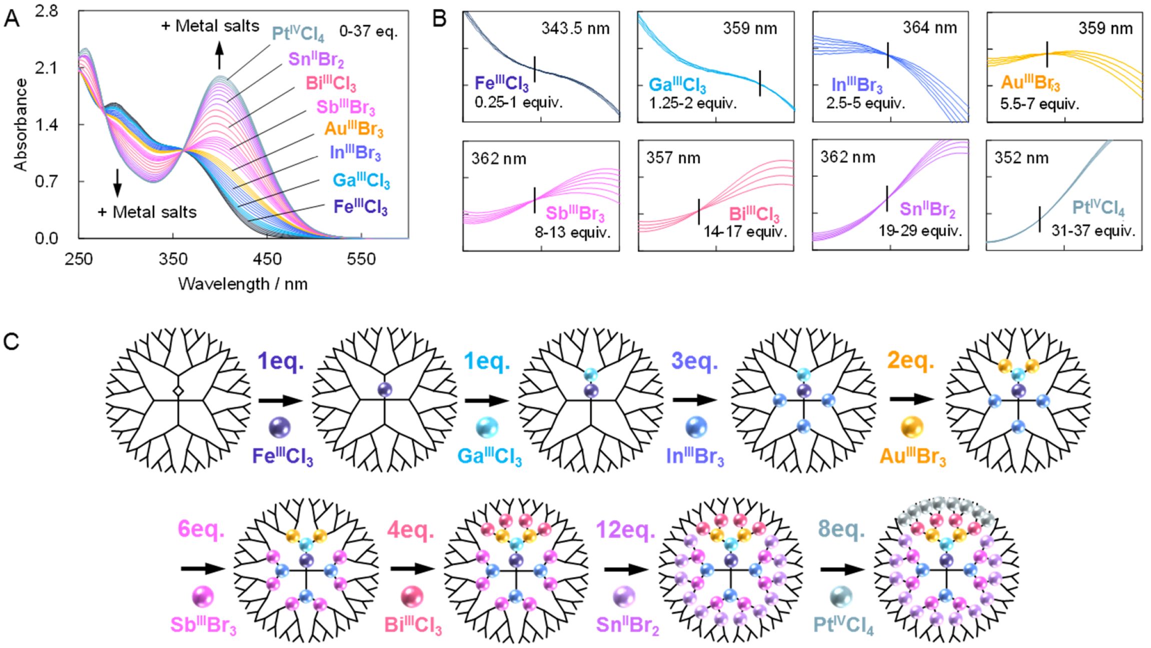

Supplementary Figure 48

Multimetallic accumulation of Fe, Ga, In, Au, Sb, Bi, Sn and Pt on PyTPM-G4. (A) Changes in UV-vis absorption spectra of PyTPM-G4 upon the addition of 1 equiv. $Fe^{III}Cl_3$, 1 equiv. $Ga^{III}Cl_3$, 3 equiv. $In^{III}Br_3$, 2 equiv. $Au^{III}Br_3$, 6 equiv. $Sb^{III}Br_3$, 4 equiv. $Bi^{III}Cl_3$, 12 equiv. $Sn^{II}Br_2$ and 8 equiv. $Pt^{IV}Cl_4$ in DCM/AN = 1:1 ([PyTPM-G4] = ca. 3.0 × 10^{-6} M, 20 °C). (B) Isosbestic points during complexation with the metal salts in respective layers 0–1, 1–2, 2–5, 5–7, 7–13, 13–17, 17–29 and 29–37. (C) Layer-by-layer stepwise multimetallic accumulation of the metal salts on PyTPM-G4.

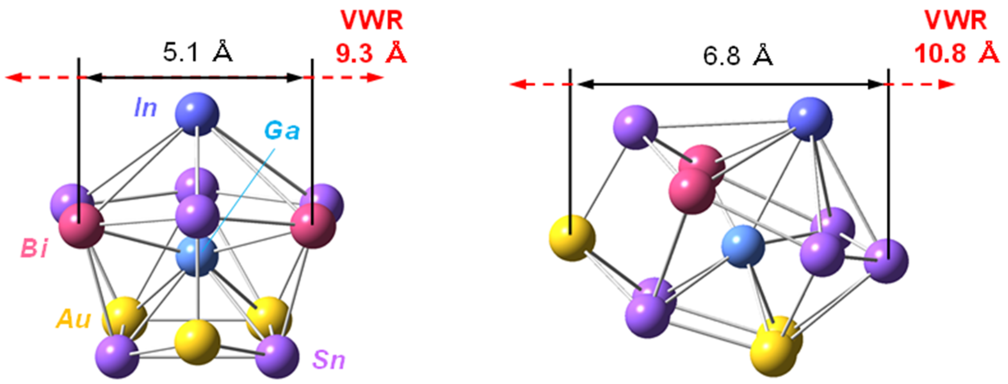

Supplementary Figure 49
One of the optimized molecular structures of the five-element cluster and its short (left) and long (right) diameters considering van der Waals radius by DFT calculation (B3LYP/LanL2DZ).

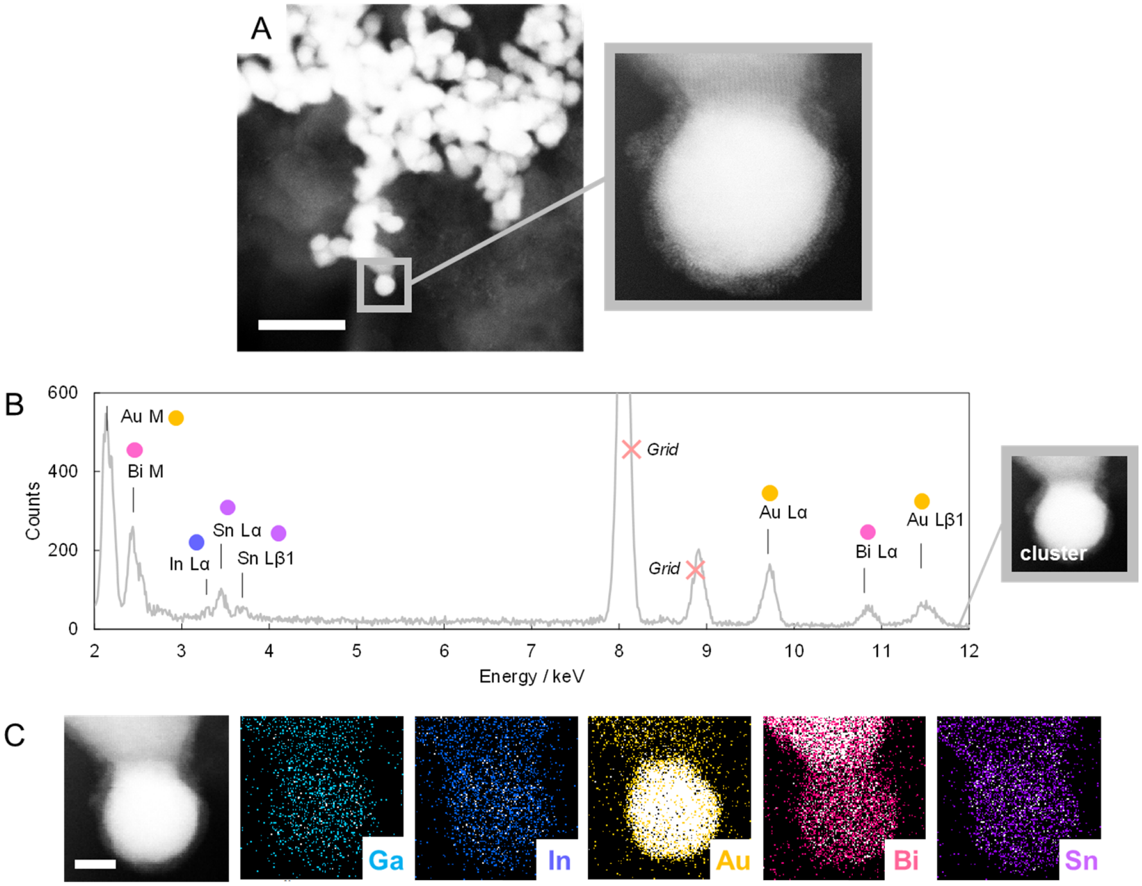

Supplementary Figure 50

The five-element cluster synthesized without the dendrimer template. (A) STEM image of the five-element cluster supported on KB synthesized in absence of dendrimer template (scale bar: 50 nm) (left) and the enlarged view of the cluster (right). (B) STEM/EDS analysis of the five-element cluster on KB synthesized in absence of dendrimer template. (C) STEM/EDS mapping of the cluster on KB (scale bar: 5 nm).

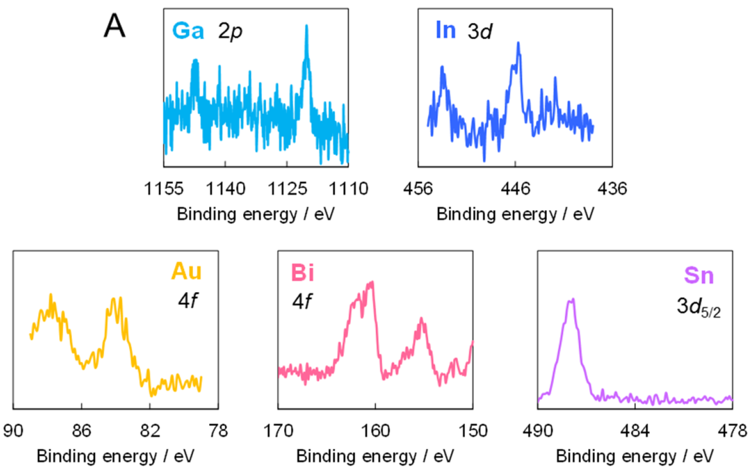

B

	Ga	In	Au	Bi	Sn
spot 1	1	1.0	0.9	0.6	5.2
spot 2	1	0.7	1.1	0.4	5.6
spot 3	1	1.2	0.9	0.5	4.8

Supplementary Figure 51

The XPS measurement of air-oxidized $Ga_1In_1Au_3Bi_2Sn_6$ sub-nanocluster on a GC substrate using X-ray beam converged into a microscopic spot. (A) XP spectra of the cluster focusing on the Ga 2*p*, In 3*d*, Au 4*f*, Bi 4*f* and Sn 3*d* core levels. (B) Position dependence of ratio of constitutive elements (Ga : In : Au : Bi : Sn) on a GC substrate. The ratio was not changed between three microscopic spots.

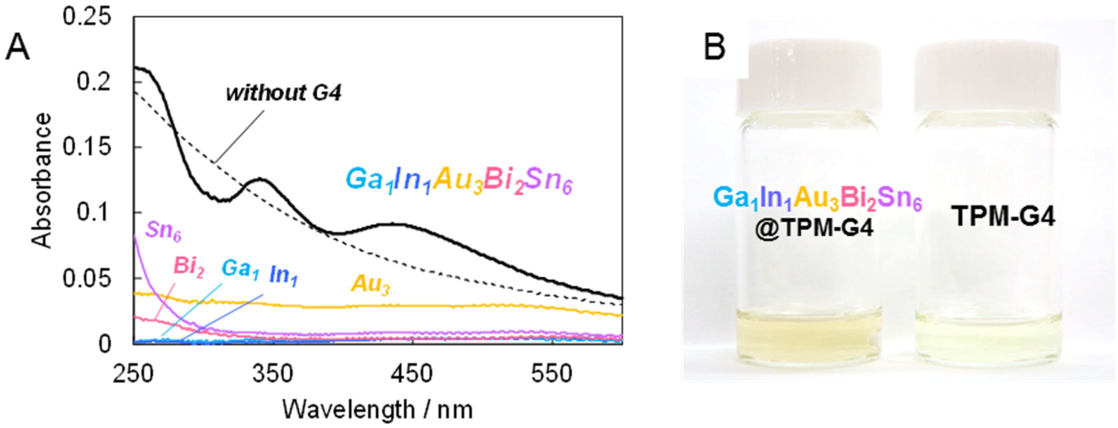

Supplementary Figure 52

(A) Solid line: UV-vis absorption spectra of $Ga_1In_1Au_3Bi_2Sn_6$, Ga_1, In_1, Au_3, Bi_2 and Sn_6 obtained by dendrimer template synthesis in DCM/AN = 1:1. The absorption of PyTPM-G4 was cancelled by the reference solution of PyTPM-G4. Dotted line: That of $Ga_1In_1Au_3Bi_2Sn_6$ synthesized without PyTPM-G4. (B) Appearances of the solutions of the five-element cluster with PyTPM-G4 (left) and only PyTPM-G4 (right).

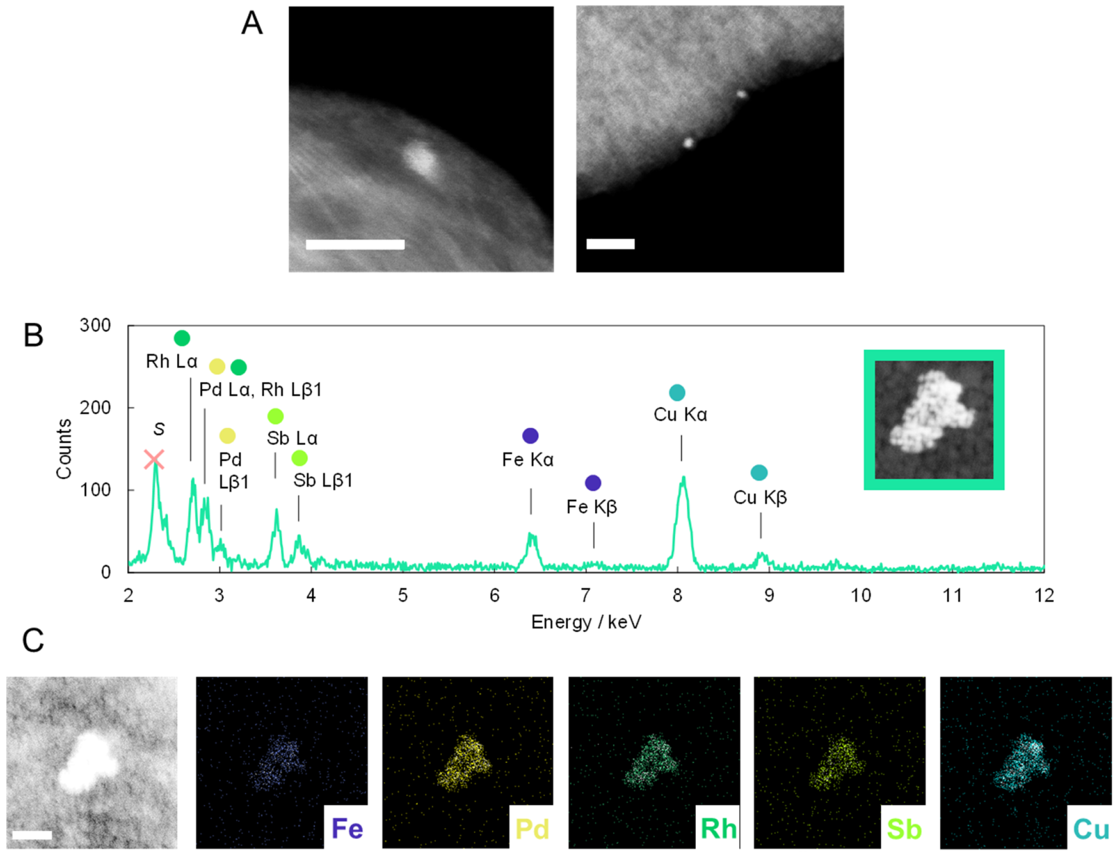

Supplementary Figure 53

The five-element sub-nanocluster $Fe_1Pd_1Rh_3Sb_2Cu_6$ synthesized by the dendrimer template. (A) STEM image of five-element cluster supported on KB (scale bar: 5 nm (left) and 10 nm (right)). (B) STEM/EDS analysis of aggregated five-element clusters on KB. Enhanced peaks by aggregation allow us to quantitate the constitutive elements. The calculated ratios of Pd : Fe : Rh : Sb : Cu of the cluster were 1 : 1.4 : 3.0 : 2.0 : 5.3 and these values are in good agreement with the theoretical ones (Pd : Fe : Rh : Sb : Cu = 1 : 1 : 3 : 2 : 6). (C) STEM/EDS mapping of the aggregated clusters on KB (scale bar: 30 nm). Each element was detected uniformly on the image of aggregates.

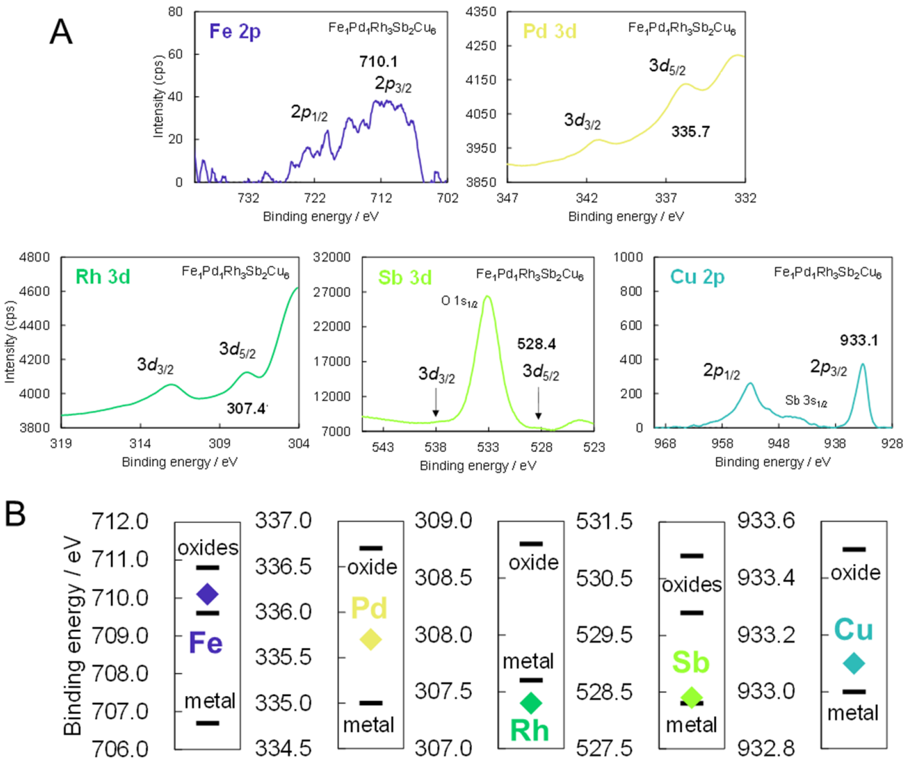

Supplementary Figure 54

The XPS measurement of five-element sub-nanocluster $Fe_1Pd_1Rh_3Sb_2Cu_6$. (**A**) XP spectra of five-element cluster, metals and metal oxides focusing on the Fe 2$p_{3/2}$, Pd 3d, Rh 3d, Sb 3d and Cu 2p core levels. (**B**) Binding energy of five-element cluster, metals and metal oxides for each element. The degree of the shifts was associated with the electronegativity (EN) of the elements (EN: Fe = ca. 1.8, Cu = ca. 1.9, Sb = ca. 2.0, Pd = ca. 2.2, Rh = ca. 2.3)[30]. Considering charge distribution depending on the EN in the molecule composed of different elements, the result also suggests that these elements are blended in the cluster. However, quantitation of constitutive elements by XPS was difficult due to overlap of the signals for each element.

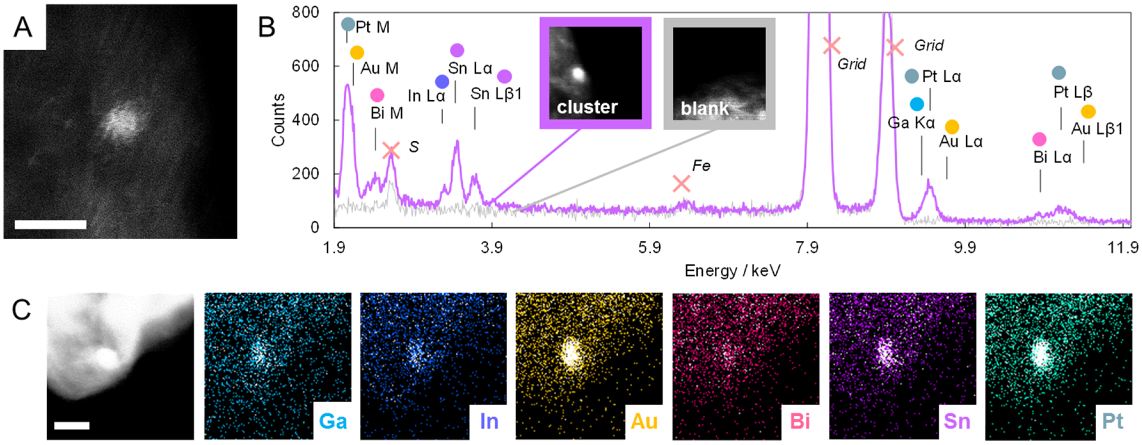

Supplementary Figure 55

The six-element sub-nanocluster $Ga_1In_1Au_3Bi_2Sn_6Pt_4$ synthesized by the dendrimer template. (A) STEM image of six-element cluster supported on KB (scale bar: 2 nm). (B) STEM/EDS analysis of six-element cluster on KB. (C) STEM/EDS mapping of the cluster on KB (scale bar: 5 nm).
